# Co-option of transcription factors drives evolution of quantitative disease resistance against a necrotrophic pathogen

**DOI:** 10.1093/plcell/koaf233

**Published:** 2025-09-30

**Authors:** Severin Einspanier, Christopher Tominello-Ramirez, Florent Delplace, Remco Stam

**Affiliations:** Department of Phytopathology and Crop Protection, Faculty of Agricultural and Nutritional Sciences, Institute of Phytopathology, Kiel University, 24098 Kiel, Germany; Department of Phytopathology and Crop Protection, Faculty of Agricultural and Nutritional Sciences, Institute of Phytopathology, Kiel University, 24098 Kiel, Germany; Department of Nutriinformatics, Faculty of Agricultural and Nutritional Sciences, Institute of Human Nutrition and Food Sciences, Kiel University, 24098 Kiel, Germany; Laboratoire des Interactions Plantes Microorganismes Environnement (LIPME), INRAE, CNRS, Castanet-Tolosan, Cedex, France; Department of Phytopathology and Crop Protection, Faculty of Agricultural and Nutritional Sciences, Institute of Phytopathology, Kiel University, 24098 Kiel, Germany

## Abstract

Wild relatives of crop species possess diverse levels of quantitative disease resistance (QDR) to biotic stresses. The genomic and regulatory mechanisms underlying these differences are poorly understood. How QDR against a generalist necrotrophic pathogen evolved and whether it is driven by conserved or species-specific regulatory networks remain unclear. We examined the transcriptomic responses of 5 diverse wild tomato species that span a gradient of QDR. We initially hypothesized that conserved regulatory modules might control QDR. We use differential gene expression analysis and weighted gene coexpression network analysis to find instead that species-specific regulatory features, encompassing both infection-induced and constitutively expressed genes, predominantly shape QDR levels. To further dissect the evolutionary basis of these regulatory patterns, we performed phylotranscriptomic analyses of gene regulatory networks. Notably, our findings reveal that the conserved NAC transcription factor 29 is pivotal in developing disease resistance only in *Solanum pennellii*. The differential regulation and altered downstream signaling pathways of NAC29 provide evidence for its co-option in the resistance mechanisms of *S. pennellii*. The role of NAC29 in conferring resistance is confirmed by the presence of a premature stop codon in susceptible *S. pennellii* genotypes. This finding highlights the species-specific rewiring of gene regulatory networks by repurposing a conserved regulatory element to effectively enhance resistance against pathogens. These results offer insights into the evolutionary and regulatory complexity underlying QDR and emphasize the significance of species-specific gene regulation in shaping resistance against a cosmopolitan necrotrophic pathogen.

## Teaser

Quantitative disease resistance in a wild tomato genotype evolved through species-specific regulatory network rewiring, with an ancient transcription factor repurposed for defense.

## Introduction

Developing or engineering durable resistance against necrotrophic pathogens in crop plants displays a major bottleneck in modern plant breeding. Although the scientific community gained a comprehensive understanding of dominant R-gene-mediated resistance, especially against biotrophic pathogens, an R-gene-mediated resistance against necrotrophic generalist pathogens like *Sclerotinia sclerotiorum* or *Botrytis cinerea* has not yet been described (Mbengue et al. 2016; Wang et al. 2019). Accordingly, the primary goal of resistance breeding against necrotrophic pathogens is to enhance quantitative disease resistance (QDR), where many independent loci contribute marginally to a continuous pattern of resistance (Caseys et al. 2021). The current state of knowledge about mechanisms involved in QDR has been synthesized in several reviews (Poland et al. 2009; Roux et al. 2014; Corwin and Kliebenstein 2017; Gou et al. 2023).

Natural or wild plant pathosystems are a valuable tool for unraveling the complexity of the QDR mechanisms and their evolution, especially when looking into crop wild relatives (Kahlon and Stam 2021). Crop wild relatives harbor a wide diversity of morphological properties and represent a common source of novel traits in modern crops (Nosenko et al. 2016; Albaladejo et al. 2017; Szymański et al. 2020). Adapting to diverse habitats is accompanied by the evolution of resistance or tolerance against numerous stresses, including drought, temperature, and disease (Bolger et al. 2014; Wei et al. 2024). For instance, previous studies have demonstrated that accessions of the crop wild relative *Solanum chilense* exhibit a wide interpopulation and intrapopulation diversity in defense response against pathogens like *Phytophthora infestans*, *Fusarium oxysporum*, and *Alternaria* spp. (Stam et al. 2017; Kahlon et al. 2021; Schmey et al. 2023). Several wild tomato species also show distinct patterns of presence–absence variation, copy number variation, and specific patterns of selection on pathogen resistance genes within and between populations or species (Stam et al. 2019; Seong et al. 2020; Silva-Arias et al. 2025). We have previously shown that wild tomato species employ distinct mechanisms to achieve QDR against *S. sclerotiorum*, particularly by modulating the duration of the asymptomatic phase (lag phase) and the speed of lesion growth (lesion doubling time, LDT). These measures appear not correlated and exhibit significant host and population specificity. Thus, species or populations might have adopted specific strategies to regulate QDR, i.e. prolonging the lag phase in one case or increasing the pathogen's LDT in another (Einspanier et al. 2024). The possibility of such differentiation is supported by population genomic analyses, showing distinct genomic separation and differentiation among *S. chilense* populations, which also exhibit diversifying elicitor responses and phytohormonal regulation (Böndel et al. 2015; Stam et al. 2017; Kahlon et al. 2023). Interestingly, a previous study investigating *Solanum pennellii* introgression lines described that coding sequence (CDS) variation in the introgressed loci did not explain the phenotypic variability and ranked regulatory variability as the main determinant of *B. cinerea* resistance on fruits, highlighting how regulatory plasticity can enable new resistance traits (Szymański et al. 2020).

The underlying regulatory interplay governing QDR remains elusive. Although recent studies identified QDR-associated quantitative trait loci, the explained phenotypic variation remains relatively low (Pink et al. 2022; Thomas et al. 2024). While the genomic characterization of QDR requires many resources (such as mapping populations, multiparent advanced generation intercross populations, or introgression lines of sufficient size) (Corwin and Kliebenstein 2017; Thomas et al. 2024), RNA sequencing (RNA-seq) is suitable for determining QDR regulation, leveraging high throughput and decreasing costs to support large-scale sampling. This enables researchers to investigate complex regulatory networks, thereby identifying and characterizing nuanced shifts in gene expression and their evolution (Gómez-Picos and Eames 2015; Delplace et al. 2020).

Gene networks are a powerful tool for characterizing relationships or interactions among genes and help understand the underlying molecular mechanisms of various phenotypes (Ovens et al. 2021; Delplace et al. 2022). Typically, gene network analyses are separated into undirected coexpression networks (like weighted gene coexpression network analysis, WGCNA) and directed gene regulatory networks (GRNs, Langfelder and Horvath 2008; Li et al. 2015). Albeit fundamentally different in the underlying statistical concepts, both GRNs and WGCNAs can be used to characterize complex regulatory networks, as previously shown in cultivated tomatoes. Tominello-Ramirez et al. (2024) integrated WGCNA and GRN to characterize a specific class of ethylene response factors (ERF) involved in defense against necrotrophic fungi of the genus *Alternaria*, illustrating how a system approach can be facilitated to describe QDR networks and their moderators. Although certain gene networks appear to be highly conserved (with some predating the existence of land plants), there is increasing evidence that GRN evolution can be a relatively quick response to different stresses (Obertello et al. 2015; Wu et al. 2021; Crow et al. 2022; Curci et al. 2022; Wei et al. 2024). Accordingly, evolutionary flexibility was hypothesized to enhance QDR robustness (Derbyshire and Raffaele 2023). However, it remains unclear how network evolution drives QDR and how phylogenic relationships determine the degree of QDR. This is mostly due to the high analytical complexity, challenging construction of cross-species networks, and multidimensional comparative analysis on nonmodel organisms exhibiting a wide range of complex phenotypes (Schoenrock et al. 2017).

The slow progress in breeding for QDR against the generalist pathogen *S. sclerotiorum*, which infects a broad range of hosts, including agronomically relevant crops such as oilseed rape, sunflower, and tomatoes, has largely been due to high conceptional complexity and technical hurdles (Derbyshire et al. 2022; Derbyshire and Raffaele 2023). Although Sucher et al. (2020) showed signs of recent gene expression acquisition and exaptation in a group of ATP binding cassette-type G transporters responding to *S. sclerotiorum* infection, the regulatory cues driving enhanced resistance through network reconfiguration against this pathogen remain unknown.

Here, we examine transcriptome dynamics and plasticity to identify both conserved and newly recruited regulators of QDR level. We employ a novel approach integrating phylotranscriptomic analysis and network inference to elucidate gene network evolution and unravel the regulatory cues driving QDR. Specifically, we investigate the regulatory architecture underlying QDR against a generalist necrotrophic pathogen, comparing the rewiring of GRNs in resistant versus susceptible genotypes across 5 tomato species (*S. chilense*, *Solanum habrochaites*, *Solanum lycopersicoides*, *S. pennellii*, and *Solanum pimpinellifolium*). We test the hypothesis that shared regulatory networks drive QDR while downstream mechanisms fine-tune species-specific regulatory responses. By integrating phylogenetic insights with transcriptomic data, we uncover patterns of gene family expansion, functional divergence, and regulatory remodeling that collectively enhance QDR.

This evolutionary approach can highlight broadly conserved gene networks and lineage-specific, rapidly diverging ones that may contribute to the partial, durable form of resistance in wild tomato relatives. Our findings offer a valuable resource that contrasts regulatory responses among genotypes with varying QDR levels, laying the groundwork for further exploration of the functional mechanisms shaping QDR.

## Results

### Wild tomatoes show signs of multiple transcriptome differentiation events

**Figure 1.**
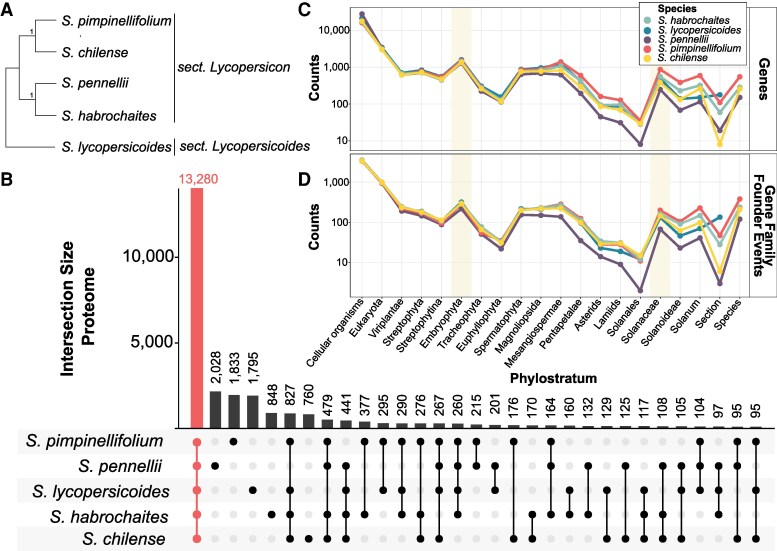
Phylogenomic analysis reveals a highly conserved proteome and recent differentiation among 5 *Solanum* species. **A)** Representative phylogenetic tree of the 5 tomato species based on BUSCO genes. Numbers represent branch support values based on quadripartition, considering the 4 clusters surrounding a branch. **B)** OrthoFinder-based UpSet plot of the proteomes, indicating that a large fraction of proteins is shared among species (highlighted in red), with only a small subset unique to individual species. Phylostratigraphic maps display the number of genes **C)** and GFFEs **D)** across the tree of life. Shaded regions highlight phylostrata corresponding to the peaks of genetic innovation during the development of land plants and within the Solanaceae. Counts correspond to the number of genes or GFFEs, respectively.

We performed a phylogenomic analysis using curated proteome sequences to corroborate the understanding of the phylogenic relationships between the 5 tomato species (*Solanum* sections Lycopersicon and Lycopersicoides): *S. chilense*, *S. lycopersicoides*, *S. habrochaites*, *S. pennellii*, and *S. pimpinellifolium* (Fig. 1A). We found that most protein sequences (13,280 orthogroups of 26,249 total) were shared among all 5 species, highlighting a strong core proteomic foundation (Fig. 1B). In contrast, species-specific unique protein sequences were relatively low, e.g. with *S. pennellii* carrying 2,028 unique proteins and *S. lycopersicoides* 1,795. This pattern underscores the evolutionary conservation within the sections while still allowing room for species-specific adaptations. To further characterize the proteomic landscape's evolutionary history, we employed phylostratigraphy to map the age of genes across all tested species.

By constructing phylostratigraphic maps, we can visualize the age of the genes and when key gene families emerged. This allows for assessing whether genes are conserved or have diversified across lineages. As anticipated, all 5 species exhibit a strongly conserved progression in the number of genes with a certain age (Fig. 1C) and the number of associated gene family founder events (GFFEs) (Fig. 1D). Most genes trace their origins back to the emergence of cellular organisms (e.g. 18,056 *S. chilense* genes from 3,346 GFFEs), with a marked decline in new gene origins over time. However, notable spikes occur during key evolutionary milestones: the development of land plants (Embryophyta, 290 GFFEs, 1,362 new genes in *S. chilense*) and the rise of flowering plants (from Spermatophyta to Mesangiospermae, in sum 2,371 new genes and 637 GFFE in *S. chilense*). Following these periods, gene assignments gradually decrease until the clade Solanaceae, where there is a renewed burst of new gene families (*S. chilense*: 144 GFFEs). All species harbor a modest number of phylostratigraphically young genes (*S. chilense*: 210 GFFEs), which may indicate ongoing species-specific differentiation or bursts of gene family innovation since the emergence of Solanaceae. However, these putatively young genes must be critically assessed, as they may partly reflect technical artifacts (such as reference genome annotation quality or analytical issues like homology detection failure).

The high number of shared orthogroups and remarkably similar age estimates for GFFEs until the clade of *Solanum* illustrate the strongly conserved evolutionary history of the 5 wild tomato species. This conservation suggests that, despite species-specific innovations, their core genomic and evolutionary trajectories have remained remarkably stable.

### *S. sclerotiorum* tolerance is highly diverse among and within wild tomato species

We previously performed a study to identify genotypes differing in disease resistance against the fungal generalist pathogen *S. sclerotiorum* (Einspanier et al. 2024). Generally, we observed a wide diversity in QDR phenotypes with strong species- and accession-specific patterns.

**Figure 2.**
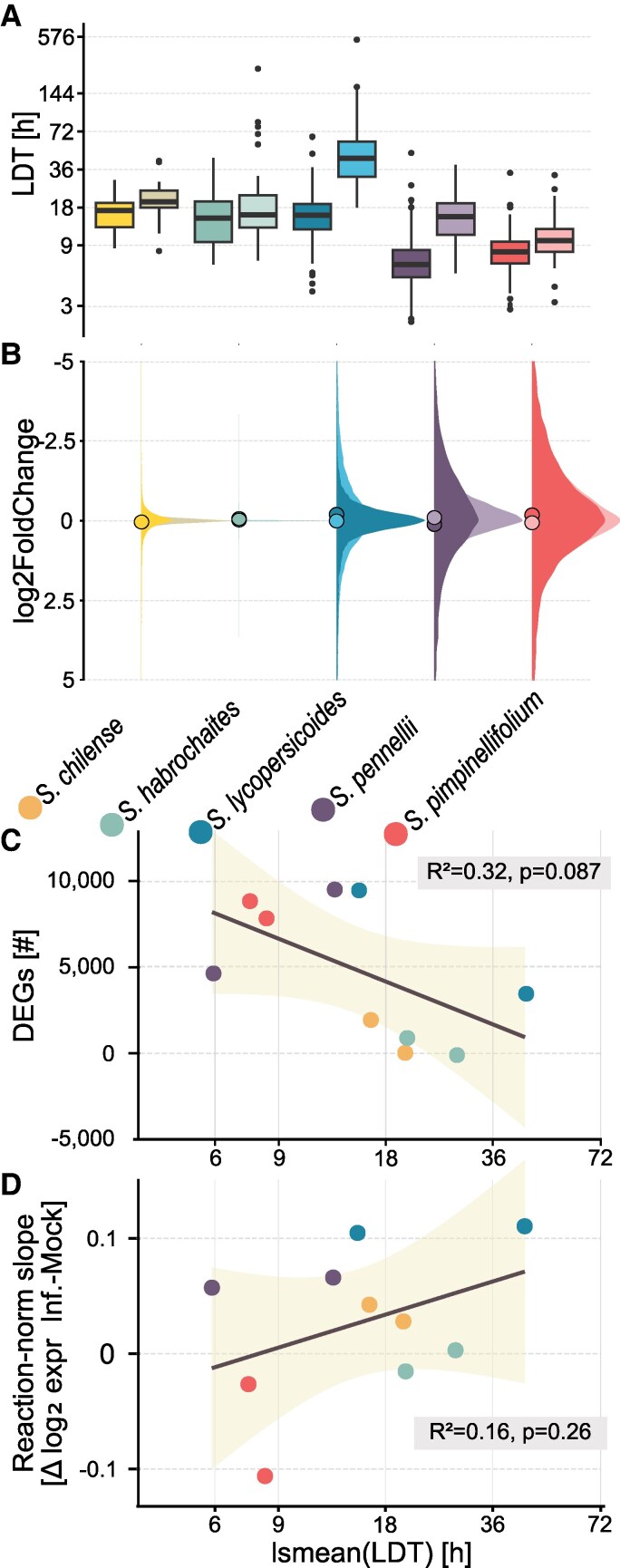
The inoculation with *S. sclerotiorum* leads to different levels of susceptibility and heterogeneous transcriptomic responses. **A)** LDT values in hours for 5 host species (2 genotypes each) serve as a quantitative measure of the level of QDR. Data adapted from Einspanier et al. (2024). **B)** Density plot of log_2_ fold change in gene expression across all accessions following infection with *S. sclerotiorum* compared with mock treatment, illustrating the overall transcriptomic response to infection. Dots represent the mean log_2_ fold change. Pearson correlation analysis testing the interaction between the number of DEGs by genotype **C)** or the mean reaction norms of gene expression **D)** with the levels of resistance (LDT). The shaded region represents the SD.

Accordingly, we observed that the lesion growth rate (denominated LDT) was generally shorter on *S. pennellii* and *S. pimpinellifolium* genotypes (i.e. pathogen growth is faster). At the same time, *S. lycopersicoides* accessions appeared relatively resistant (Fig. 2A). We found the biggest quantitative differences between genotypes of the same species for *S. lycopersicoides* (lsmean_susceptible_ = 6.80 h vs. lsmean_resistant_ = 7.88) and *S. pennellii* (lsmean_susceptible_ = 5.86 h vs. lsmean_resistant_ = 6.65 h, see Supplementary Table S1).

### *S. sclerotiorum* infection-induced shift in gene expression underlies genotype specificity

We then conducted gene expression profiling on detached leaves during the lesion growth phase, contrasting accessions with different LDTs. An average of 4.3 mio. reads mapped to the respective reference genomes, with a mean mapping rate between 74% and 89% (mapping statistics in Supplementary Table S2, Fig. S1). We performed differential gene expression analysis by genotype comparing infected versus control conditions. Interestingly, we observed strong variation in differential (infection-induced) gene expression levels between the species. We observed few weakly differentially expressed genes (DEGs) for both *S. chilense* (sus. genotype: 2,104 DEGs, res. genotype 194 DEGs, Fig. 2B). and *S. habrochaites* genotypes (sus. genotype: 1,058 DEGs, res. genotype 65 DEGs) and a stronger regulatory response by the other host species (*S. pennellii*: sus. genotype: 4,808 DEGs, res. genotype 9,685 DEGs; *S. lycopersicoides*: sus. genotype: 3,627 DEGs, res. genotype 9,635 DEGs; and *S. pimpinellifolium*: sus. genotype: 9,007 DEGs, res. genotype 8,007 DEGs, see Supplementary Table S3). We performed a Pearson correlation analysis to test the relationship between the level of QDR and the absolute number of DEGs and observed a nonsignificant, negative association between the 2 parameters (*P* = 0.087, *R*^2^ = 0.32, Fig. 2C). Also, no significant correlation was found between the regulatory plasticity (as reaction norms) and LDT (*P* = 0.026, *R*^2^ = 0.16, Fig. 2D). However, nested species effects with contrasting trends might not be reflected sufficiently by this analysis, as we measured a lower number of DEGs on the resistant *S. lycopersicoides* genotype versus the susceptible genotype and the opposite trend for *S. pennellii.* These findings suggest that the absolute number of genes reprogrammed during infection alone does not explain QDR variability at this level. We also found no relationship between sequencing/mapping depth and the number of DEGs (see Supplementary Fig. S1).

 

### Genotypes of the same species show strong regulatory plasticity

**Figure 3.**
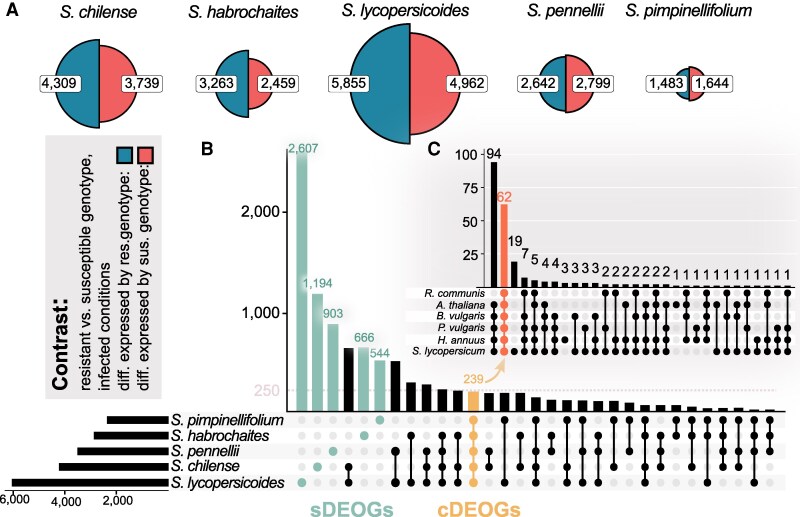
Species-specific differential gene expression related to varying QDR levels. **A)** Pie charts showing the number of genes differentially regulated between genotypes with varying levels of QDR within each species. DEGs were defined between genotypes with differing levels of QDR in infected conditions. **B)** UpSet plot illustrating the overlap of between-genotype differentially expressed orthogroups across the 5 tomato species. This intersection distinguishes species-specific differentially expressed orthogroups (sDEOGs) from a set of shared core differentially expressed orthogroups (cDEOGs). **C)** UpSet plot presenting the number of differentially expressed cDEOGs observed in other Pentapetalae species upon *S. sclerotiorum* infection, highlighting the conservation and variability of these core genes across a broader phylogenetic context. Data adapted from Sucher et al. (2020).

Seeing the results above and knowing that constitutively expressed defense-associated genes might also govern QDR, we performed a differential gene expression analysis contrasting the resistant versus the susceptible genotype by species in infected conditions. This allows the identification of infection-induced regulatory features and the expression of noninduced genes alike. Interestingly, the number of DEGs between the 2 genotypes varies strongly between the species. While we found strong alterations in the magnitude of differential gene expression between 2 *S. lycopersicoides* genotypes (5,855 sign. up-regulated genes in the resistant genotype, 4,962 downregulated, respectively), *S. pimpinellifolium* genotypes differed on a much smaller scale (1,483 up-regulated DEGs, 1,644 downregulated DEGs). Intermediate levels of differential expression between *S. pennellii* genotypes, in contrast to strong differences in *S. lycopersicoides*, clearly illustrate that the order of magnitude in gene expression does not explain the level of resistance or susceptibility (Fig. 3A).

Next, we tested whether resistance or susceptibility DEGs are unique or shared among species. For this, we selected all DEGs between resistant and susceptible accessions within a species and conducted a membership analysis based on gene orthologs to classify differentially expressed orthologs (DEOGs). We observed that most DEOGs were unique to the respective species. Accordingly, we measured 2,607 specific DEOGs (sDEOGs) for *S. lycopersicoides*, 1,194 sDEOGs for *S. chilense* and 903 sDEOGs from *S. pennellii.* However, we also identified 239 core DEOGs (cDEOGs) shared among all 5 tomato species, indicating that those genes are differentially expressed between the 2 genotypes of all species (Fig. 3B). Interestingly, we could show that the majority of those cDEOGs is differentially expressed upon *S. sclerotiorum* inoculation in 6 further related Pentapetalae plants: 62 of the cDEOGs are differentially expressed in all tested plant species (*Ricinus communis*, *Arabidopsis thaliana*, *Beta vulgaris*, *Phaseolus vulgaris*, *Helianthus annuus*, and *Solanum lycopersicum*), and 94 cDEOGs in all plants of this set excluding *R. communis.* We further identified 19 cDEOGs, which might be specific to the genus *Solanum* (Fig. 3C). These findings illustrate that only a small proportion of all DEOGs show a conserved expression pattern over Pentapetalae plants. At the same time, regulatory differentiation between genotypes with contrasting QDR appears to be mostly specific to the nested species.

In the Pentapetalae plant set, most conserved differentially expressed orthologous genes (cDEOGs) are significantly induced by infection. However, within our wild tomato dataset, only a small fraction of these genes (*n* = 16) is both infection-induced and vary significantly between genotypes of differing QDR. Notably, most cDEOGs exhibited pronounced presence–absence patterns in their infection response (see Supplementary Fig. S2), suggesting that their regulation is highly variable across species. Species-specific DEOGs also display differing regulatory profiles: While ∼60% of *S. pimpinellifolium*, *S. pennellii*, and *S. lycopersicoides* sDEOGs are induced by infection, only a marginal fraction of sDEOGs in *S. chilense* or *S. habrochaites* is induced (Supplementary Fig. S2). This pattern underscores the regulatory flexibility and diversity among wild tomato species in their response to pathogenic infection, likely reflecting a wide range of adaptive strategies in their defense mechanisms (Supplementary Tables S4 to S8).

### The regulation of induced cDEOGs is diversified among *Solanum* species

**Figure 4.**
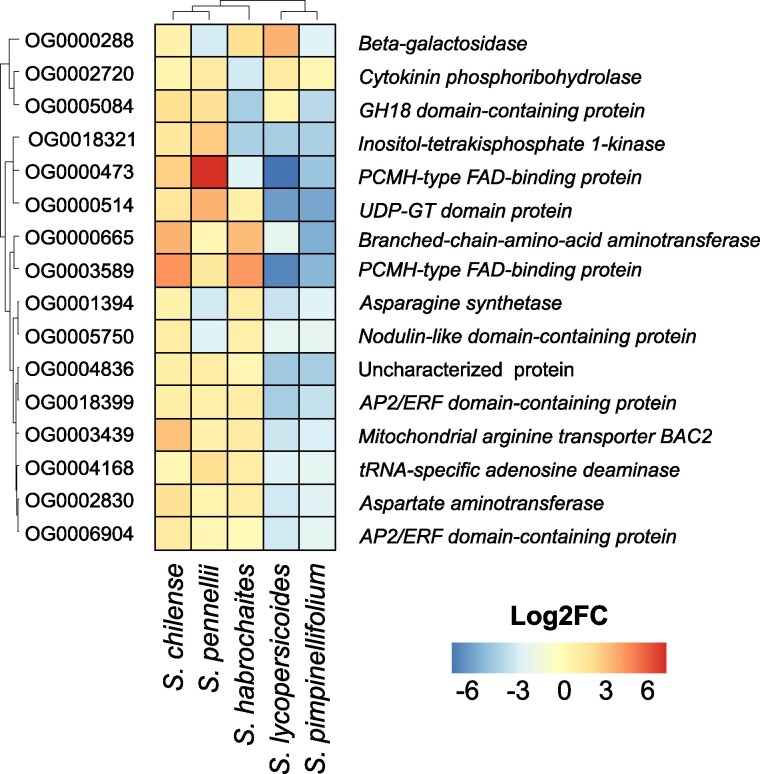
Differential expression of 16 core infection-induced DEOGs. The heatmap illustrates the differential expression levels of 16 core infection-induced DEOGs across the 5 tomato species. Each row represents a gene, and columns correspond to comparisons between resistant and susceptible genotypes within each species. The colors indicate the magnitude and direction of expression differences in infected conditions, showing inconsistent gene regulation patterns associated with resistance and susceptibility to infection.

We characterized the function of the 16 infection-induced cDEOGs (icDEOgs) (see Supplementary Table S9). Interestingly, we identified 2 genes (OG0006904 and OG0018399) matching transcription factors (TFs) of the AP2/ERF family. One of them, OG0018399, was previously characterized as an *S. lycopersicum* ERF-D6 TF (Tominello-Ramirez et al. 2024). Furthermore, we identified DEOGs encoding a putative chitinase/cell-wall degrading enzyme (OG0005084) and a putative UDP-glycosyltransferase (OG0000514) among oxidative stress regulation (Fig. 4).

We hypothesized that the expression pattern of those infection-induced cDEOGs might be mostly uniform, as they are induced by infection and differentially regulated between susceptible and resistant genotypes. However, we observed a diversified regulatory pattern among all induced cDEOGs with contradictory regulation. Most interesting, the putative ERF D6 (OG0018399) is significantly upregulated in the resistant genotypes of *S. habrochaites*, *S. pennellii*, and *S. chilense*. Yet, it is downregulated in the resistant genotypes of *S. pimpinellifolium* and *S. lycopersicoides* (indicating a higher induction in the susceptible genotypes, Fig. 4). Interestingly, except for OG0004836, all induced cDEOGs share an evolutionary origin predating the emergence of flowering plants, suggesting that these highly conserved genes remain subject to differential regulation (Supplementary Table S9). Concluding, our differential gene expression (DEG) analysis gained initial insights into the regulatory dynamics driven by contrasting infection conditions and/or genotypes and shows that rewiring of expression pattern of shared infection-induced DEOGs may be source of QDR adaptation at the species level.

### WGCNA reveals evidence of transcriptome specification

While informative, DEG analysis alone cannot fully capture the complexity of the underlying regulatory pathways and networks. Thus, to further investigate global transcriptional rewiring of QDR responses in our 5 species and to achieve a more comprehensive understanding of QDR across species, we employed multiple WGCNA.

**Figure 5.**
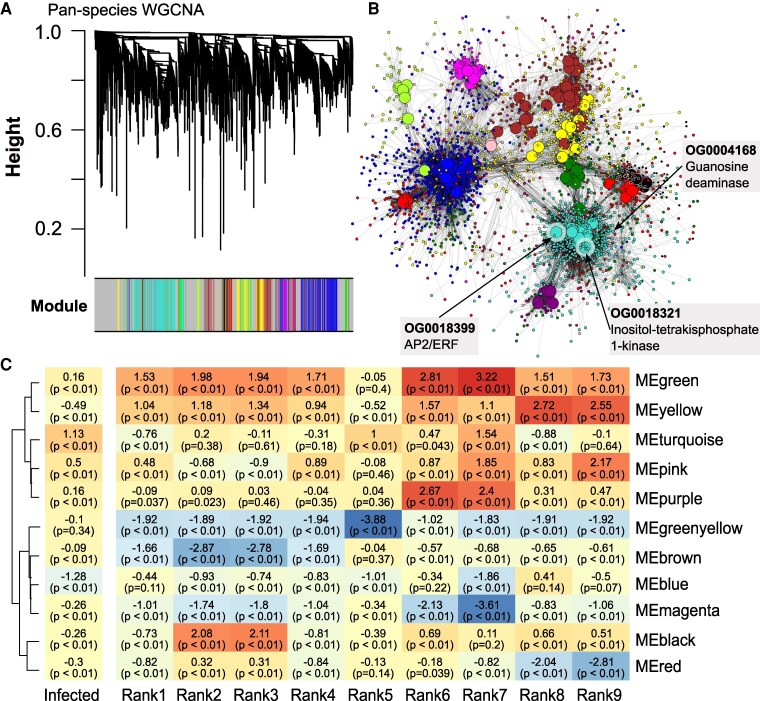
Weighted gene correlation analysis of single-copy orthogroups across 5 tomato species. **A)** Dendrogram illustrating the clustering of single-copy orthogroups into modules, with each module assigned a unique color. **B)** Network visualization of the orthogroup-based modules, where large nodes represent highly connected hub genes. Orthogroup IDs and protein names of infection-induced cDEOGs identified in previous analyses are highlighted with boxes. **C)** Module–trait relationships derived from a linear model using MEs as the response and infection state plus resistance rank as predictors. Resistance rank is relative to the most resistant genotype (*S. lycopersicoides*, LA2951), and infection status is compared with mock conditions (see Supplementary Table S1). The coefficients shown represent effect size estimates from ANOVA, while the accompanying *P*-values indicate significance after FDR correction.

First, we constructed an orthology-based pan-species network. We hypothesized that the overarching gain of QDR might be correlated with the module eigengenes (MEs). To test this, we constructed a gene-correlation network based on 7,419 single-copy orthogroups, which were grouped into 11 distinct coregulatory modules, with a total of 462 hub genes (Fig. 5, A and B, Supplementary Fig. S3). Only 3 core infection-induced DEOGs were found in this network (Fig. 4B, Supplementary Table S10). Following network construction, we examined both intermodular and intramodular edges. We validated the biological relevance of module assignments through functional assignments using Gene Ontology (GO) terms. We used a linear model to infer QDR phenotypes as ranks with the MEs.

Interestingly, we found no significant and biologically meaningful association of ME expression with the LDT. While certain modules (e.g. blue, yellow, turquoise) showed a strong association with the infection as such, there was no clear gradient in ME estimates from the most susceptible to the most resistant genotype. Moreover, despite having similar LDT (Rank7/Rank8, Supplementary Table S1), these 2 genotypes show notably contrasting MEs in infection-associated modules (for instance, Rank7 blue = −1.86 vs. Rank8 blue = 0.41). At the same time, the strongest associations in the module–trait relationships emerge between genotypes within the same species (such as Rank2 and Rank3 or Rank1 and Rank4), suggesting that QDR regulation could be tightly integrated into genotype- or species-specific regulatory frameworks (Fig. 5C, Supplementary Table S11).

Accordingly, we hypothesize that the OG-based network (based on 2 genotypes per species) may lack the statistical power to link regulatory patterns to cross-species phenotypes confidently or that phenotypic resistance is achieved through interlinked, species-specific coregulatory modules.

### Species-specific network topology is linked to QDR variation

**Figure 6.**
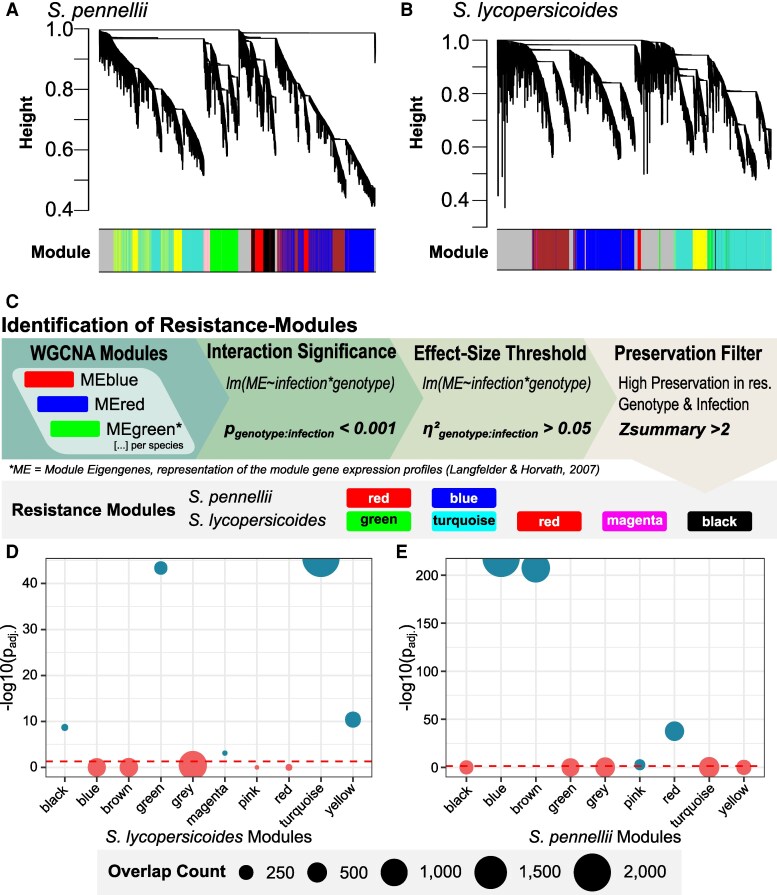
WGCNA of **A)**  *S. pennellii* and **B)**  *S. lycopersicoides* RNA-seq data. Candidate resistance modules were selected using a 3-step classification pipeline **C)**. First, we tested the significance of each ME on the infection:genotype interaction using a linear model. Modules with a significant interaction term were subsequently filtered based on effect size (*η*^2^), followed by filtering for high preservation in the resistant genotype when infected (*z*-summary > 2). This analysis was applied to all MEs across all tested species. Resistance-associated modules of *S. pennellii* and *S. lycopersicoides* are highlighted. To characterize the overlap of genes in putative *S. pennellii*  **D)** and *S. lycopersicoides*  **E)** resistance modules, we performed a membership analysis using orthogroup-based inference and Fisher's exact test. The dot size indicates the number of overlapping genes, while the dashed line indicating the threshold for a significant enrichment.

We, therefore, performed per-species regulatory network analyses to gain higher-resolution insights into QDR regulation. Specifically, we compared the WGCNA modules of *S. lycopersicoides* and *S. pennellii*—the 2 species that displayed the most pronounced differences in resistance phenotypes across the tested genotypes. In *S. pennellii*, 16,577 genes were clustered into 8 coexpression modules, while 18,558 *S. lycopersicoides* genes were grouped into nine modules (Fig. 6, A and B). We used a custom pipeline to identify co-regulatory modules potentially involved in the development of resistance. In short, we selected modules with a significant genotype × infection interaction, a substantial effect size and high preservation in infected, resistant genotypes (Fig. 6C and Materials and methods section). Using this approach, we identified 2 resistance modules in *S. pennellii* (“red” and “blue”) and 5 in *S. lycopersicoides* (“black,” “red,” “turquoise,” “green,” and “magenta,” Fig. 6C, Supplementary Tables S12 and S13). Next, we combined both datasets using orthogroups to quantify the extent of overlap between resistance modules of the respective species. We assessed the robustness of this overlap using Fisher's exact test with false discovery rate (FDR)-corrected *P*-values. 2,460 of 3,683 orthologous *S. pennellii* resistance genes were significantly enriched in the *S. lycopersicoides* resistance modules (Supplementary Tables S14 and S15, Fig. 6, D and E). However, a significant number of *S. pennellii* genes did not cluster in *S. lycopersicoides* resistance modules: 456 genes were assigned to the blue module and 479 to the brown module. Interestingly, 1,177 genes from the *S. pennellii* resistance modules could not be assigned to a specific *S. lycopersicoides* module and were assigned to the gray module. We observed a similar pattern when projecting genes from the *S. lycopersicoides* resistance modules onto the *S. pennellii* GRN. In this case, 2,449 of the 3,702 orthologous genes were located in the *S. pennellii* resistance-associated blue module. In comparison, a significant number of genes (1,121 genes) was assigned to the brown module, which is specific to infection response rather than resistance. These findings indicate that although several QDR-related genes might be shared across species, a significant subset of QDR genes remains associated with distinct nonresistance modules reflecting species-specific regulatory fine-tuning.

### Functional enrichment reveals a broad core functional set and specific species functions

**Figure 7.**
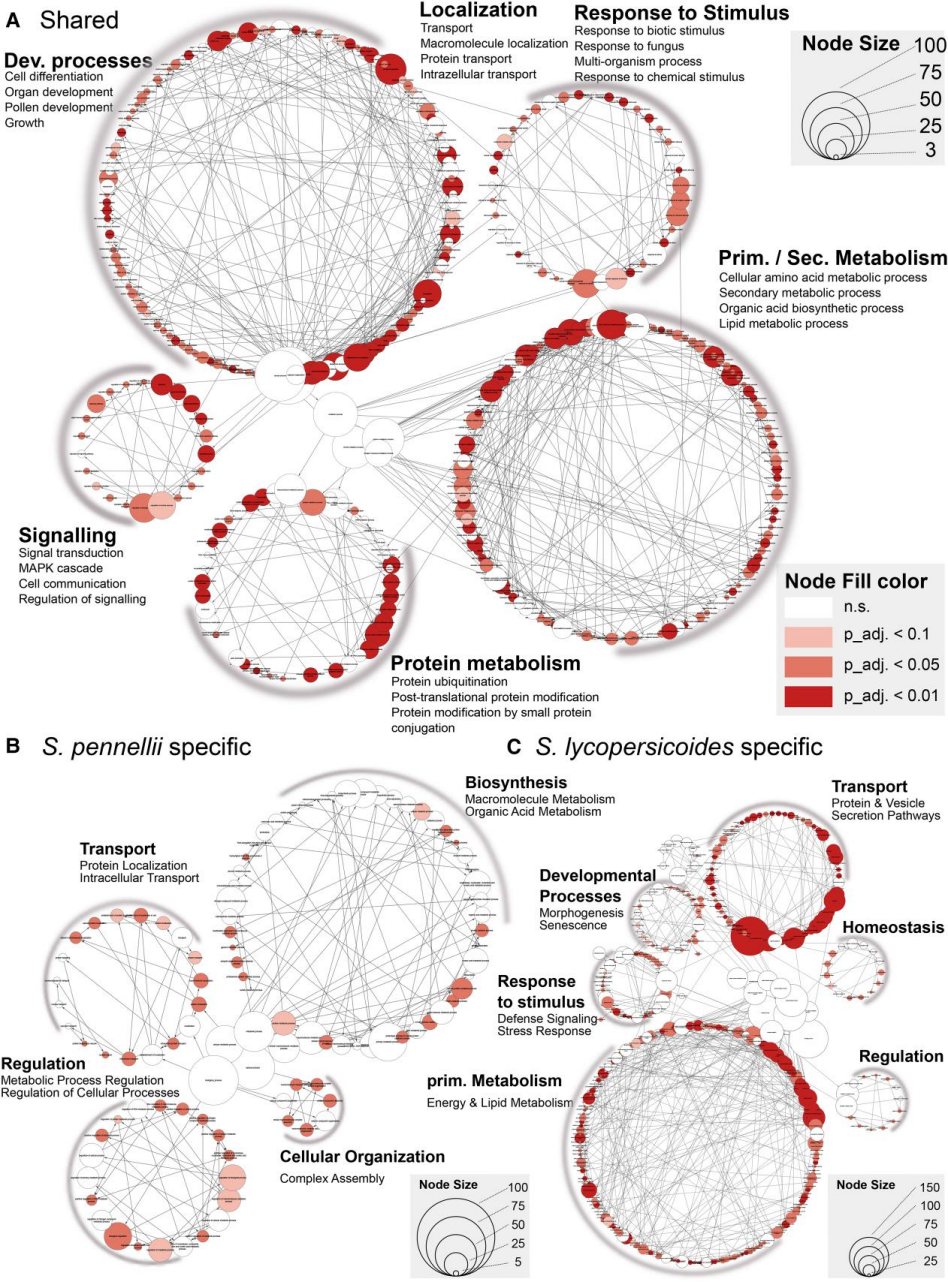
GO enrichment analysis of resistance coexpression modules. **A)** Core genes shared across the resistance modules of *S. pennellii* and *S. lycopersicoides.*  **B)**  *S. pennellii*-specific genes, **C)**  *S. lycopersicoides-*specific genes. In each panel, the size of a dot corresponds to the number of genes associated with a GO term, while the fill color indicates the FDR-corrected significance level of the enrichment. This visualization highlights key functional categories enriched in core and species-specific resistance responses.

We then performed a GO term enrichment analysis on both species' shared and unique QDR-module gene sets to assess whether those harbored specific functional traits. Among the 2,460 overlapping genes, we identified a diverse array of significantly enriched GO terms clustered into 6 groups: primary/secondary metabolism, localization, developmental processes, protein metabolism, signaling, and response to stimulus. Within these clusters, we found enrichment of protein transport and localization, lipid and carbohydrate metabolism, and cinnamic acid synthesis involving processes. Notably, we did not observe a clear enrichment for hormone homeostasis (Fig. 7A). In contrast, the *S. pennellii*-specific genes were clustered into 4 functional groups: biosynthesis (including macromolecule/protein glycosylation), positive regulation of cellular/biological processes, stress response, and catabolism, with modest enrichment for signaling functions (Fig. 7B).

For *S. lycopersicoides*, we identified nine differentially regulated functional clusters, with a particular emphasis on transport, amino acid metabolism, general metabolism, and defense signaling (Fig. 7C). These findings support a 2-tier model of QDR regulation between species: a broad, shared set of genes provides general disease resistance, while host-specific mechanisms offer more targeted functions. For example, *S. pennellii* may rely on detoxification via glycosylation, whereas *S. lycopersicoides* could depend more on specialized transport processes. This model underscores how an equilibrium of shared and species-specific strategies may shape effective defense responses across different *Solanum* species.

### Shared QDR regulation compromises evolutionarily conserved genes

To test the hypothesis that the specification of QDR signaling may be subject to diversifying selection, we calculated the transcript age index (TAI) and transcript divergence index (TDI) for both shared and species-specific gene sets. Our analysis revealed that species-specific genes are evolutionary younger, as suggested by increased TAI (e.g. *S. pennellii*: overlap TAI = 1.69 vs. unique TAI = 1.93; *S. lycopersicoides*: overlap TAI = 1.73 vs. unique TAI = 2.36, see Table 1). Additionally, shared genes from resistance modules showed lower TDI values, indicating signs of stronger purifying selection than observed on the unique sub-modules (*S. pennellii*: 4.3 vs. 4.7; *S. lycopersicoides*: 4.3 vs. 4.9, see Supplementary Table S16). This is further supported by significantly elevated ratio of nonsynonymous mutations to synonymous mutations (*dN/dS*) in each species' uniquely regulated gene set compared with the whole resistance modules (Supplementary Fig. S4). Accordingly, we hypothesize that both species share a conserved, ancestral suite of genes that presumably form the “backbone” of the quantitative defense network regulation, while younger, less strongly conserved genes underlie specification. This balance between conservation and innovation might be critical for the dynamic fine-tuning of defense responses against generalist pathogens over evolutionary timescales.

**Table 1.** Per-species TAI of resistance modules covering shared and unique gene sets

*Species*	*Whole resistance modules*	*Overlap*	*Unique*
*S. pennellii*	1.769 ± 0.006	1.690 ± 0.008	1.931 ± 0.008
*S. lycopersicoides*	1.994 ± 0.015	1.736 ± 0.010	2.365 ± 0.023

Mean TAI values are provided with ±SD.

### QDR in *S. pennellii* genotype LA2963 may have evolved by co-option of a TF

To test the above hypothesis, we conducted a gene-regulatory network analysis to determine whether the TFs or the periphery drive the evolutionary signature of the network. We extracted potentially interesting TFs according to the following criteria: (i) hub in the WGCNA network (high connectivity), (ii) gene in the resistance module, and (iii) hub in the GRN and identified highly connected TFs for both focal species (18 in *S. lycopersicoides* and 10 in *S. pennellii*). We then focused on TFs exhibiting differential expression patterns in response to infection and between genotypes with varying resistance levels.

**Figure 8.**
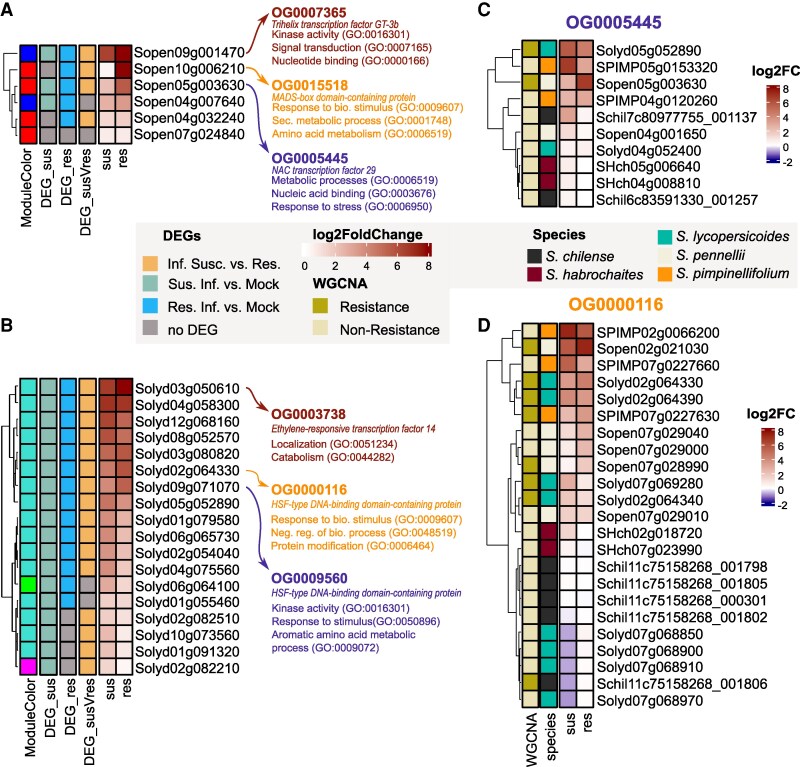
GRN analysis reveals a shared TF co-opted for divergent regulation and coexpression. The heatmaps in **A)** and **B)** illustrate TF regulation in susceptible and resistant genotypes of *S. pennellii* and *S. lycopersicoides*, respectively. Each TF is a hub gene in the GRN and WGCNA and is located in a resistance module. Each heatmap row corresponds to a TF, with the “ModuleColor” column indicating its assignment to a specific WGCNA resistance module. Differential expression analysis of relevant contrasts is also displayed, with the 3 most strongly regulated TFs highlighted along with their corresponding orthogroups. For these key TFs, summarized GO terms for their downstream coexpressed genes are provided, offering insights into their functional roles in resistance pathways. The regulation of these resistance-associated TFs **C)**  *S. pennellii:* Sopen05g003630-OG0005445, **D)**  *S. lycopersicoides: Solyd02g064330-OG0000116*) was then compared across species using orthogroups. The expression of each gene within the respective orthogroup was visualized across varying levels of QDR (susceptible|resistant), with color coding indicating the respective species and the assignment of each TF to its species-specific resistance module.

In *S. pennellii*, this subset includes a putative Trihelix TF GT-3b (Sopen09g001470, OG0007365), a MADS-box domain containing-protein (Sopen10g006210, OG0015518) and NAC TF 29 (Sopen05g003630, OG0005445, Fig. 8A). In *S. lycopersicoides*, we observed an ethylene-response TF 14 (Solyd03g050610, OG0003738), and 2 HSF-type DNA-binding domain-containing proteins (Solyd02g064330, OG0000116, Solyd09g071070, OG0009560) with a strong association with the resistant genotype (Fig. 8B, Supplementary Table S17). Generally, these TFs were more strongly induced in the resistant genotype and are part of a resistance-associated coregulatory module.

We then examined whether the orthologs of these putative resistance-related TFs showed similar patterns in the other species. We discovered a wide diversity in TF expression among all members of the same orthogroup. For example, the TF Sopen05g003630 (OG0005445) was strongly induced only in the resistant genotype of *S. pennellii*, although this gene is present and expressed in other species. Moreover, OG0005445 is not specifically induced in resistant genotypes nor assigned to a resistance module in most of the other species. This suggests that the regulation of Sopen05g003630 (OG0005445) might represent a resistance mechanism unique to *S. pennellii*. So, the regulation and the downstream processes of this TF might have shifted in *S. pennellii* compared with the other species (Fig. 8, A and C). Unique single nucleotide polymorphisms (SNPs) in the 2-kb promoter region of *S. pennellii* NAC29, along with a premature stop codon in the CDS of a highly susceptible *S. pennellii* genotype, further strengthen the evidence for a functional role of NAC29 in mediating resistance in this species (Supplementary Figs. S5 and S6, Data Sets 1 to 6). The genes Sopen10g006210 (OG0015518) and Sopen09g001470 (OG0007365) show an increased expression in the resistant *S. pennellii* genotype. While we could not detect a strong expression of Sopen10g006210 in the other species, the expression pattern of Sopen09g001470 is not linked to the QDR phenotypes (Supplementary Fig. S7). We observed a similar pattern of diversifying regulation on the *S. lycopersicoides* genes Solyd03g05610 (OG0003738) and Solyd09g071070 (OG0009560), with no clear interspecific QDR-regulation (Supplementary Fig. S7).

In contrast, the *S. lycopersicoides* gene Solyd02g064330 (OG0000116) appeared highly conserved across species with high similarity in expression pattern among the other species (orthologs also belong to resistance modules and are more highly expressed in resistant genotypes). Even so, we found strong differential regulation depending on the individual paralog (Fig. 8D). Interestingly, low *dN*/*dS* ratios suggest that both TFs, Sopen05g003630 and Solyd02g064330, are under purifying selection (Solyd02g064330 *dN*/*dS* = 0.4539 and Sopen05g003630 *dN*/*dS* = 0.1884) (Supplementary Table S18). Additionally, the GRNs of all resistance TFs are enriched for younger genes that emerged with the flowering plants and show a significant under-representation of older genes compared with the entire transcriptome (Supplementary Fig. S8). These younger genes and the low *dN*/*dS* ratios do not fully explain the novel regulatory functions. Therefore, we propose that regulatory evolution, rather than changes in coding sequences, drives the evolution and short-term adaptation of these TFs.

## Discussion

In this study, we asked whether variance in observed QDR phenotypes against generalist pathogens in closely related yet diverse species is governed by 1 or multiple mechanisms and how such variance can evolve. Singular studies on gene or gene family evolution alone cannot fully capture this complexity (Kahlon and Stam 2021). Therefore, we performed integrated transcriptomics across 5 wild tomato species infected with *S. sclerotiorum*. By combining differential expression, WGCNA, GRN, and deeper evolutionary analysis, we uncover both conserved and species-specific QDR strategies, highlighting a complex interplay between core and adaptive regulatory mechanisms.

### Resistance against *S. sclerotiorum* underlies a complex regulatory interplay

Studies on diverse crops and model plants (e.g. soybean, rapeseed, sunflower, bean, pepper, tomato, *A. thaliana*) reveal broad variation in QDR against *S. sclerotiorum* (Boland and Hall 1994; Yanar and Miller 2003; Fusari et al. 2012; Mei et al. 2012; Uloth et al. 2013; Boudhrioua et al. 2020; Chauhan et al. 2020; Sucher et al. 2020; Ding et al. 2021; Mazumdar 2021; Musa-Khalifani et al. 2021; Einspanier et al. 2024). Accordingly, QDR may be governed by a complex array of regulatory and genomic features, as suggested by the large variation in DEGs, ranging from a few dozen to over 20,000, observed under diverse experimental conditions (Joshi et al. 2016; Wu et al. 2016; Fass et al. 2020; Sucher et al. 2020; Chen et al. 2022; Tang et al. 2023). However, our findings highlight that neither the number of DEGs following *S. sclerotiorum* infection nor the regulatory plasticity is directly linked to QDR levels within or between species. In a broad-scale study, Sucher et al. (2020) proposed that regulatory flexibility and specificity of gene expression changes are essential for QDR. We show that the differential expression of resistance-associated genes is highly divergent between the species, and shared genes show contrasting regulatory patterns among the species. The wide variability of DEG counts and -regulation, as well as the dominance of species-specific patterns, underscores the complexity of plant-pathogen interactions and suggests the role of distinct molecular strategies.

### QDR-regulation is based on both host-specific and conserved genes

We examined both interspecific and intraspecific regulatory responses to infection. Comparing gene regulatory and coexpression networks across species remains challenging, and cross-species mapping does not allow to identify species-specific genes. Therefore, we directly compared DEGs and network topologies by focusing on orthologous genes (Langfelder et al. 2011; Külahoglu et al. 2014; Hu et al. 2016; Ovens et al. 2021). This approach preserves a larger number of species-specific transcripts and facilitates the projection of findings onto more distantly related plant species. Furthermore, leveraging orthologs maintains the integrity of each species' unique transcriptome and enhances our ability to uncover both conserved and unique regulatory mechanisms that underpin similar phenotypic outcomes.

Although the tested genotypes share notable genome/proteome similarity, the regulatory networks remain highly species-specific, suggesting that fine-scale shifts in gene regulation may drive the emergence of unique traits (Smith et al. 2014; Halfon 2017). In some cases, genotype effects surpass those induced by infection, suggesting genotype-specific signaling dominates in certain backgrounds. This aligns with our WGCNA-based observation that regulatory patterns correlate more strongly with species identity than any uniform resistance gradient. Nevertheless, each species possesses co-regulatory modules linked to resistance. Focusing on the overlap between *S. pennellii* and *S. lycopersicoides*, we found that shared modules appear involved in common defense responses. In contrast, unique sub-modules focus on specialized traits (e.g. signaling or detoxification). This suggests that GRNs are finetuned on a species level, also when it comes to defense against broad-host-range necrotrophic pathogens (Smith et al. 2014). Other indications of specialization of GRNs for supposedly generic tasks exist: only a minority of *S. lycopersicum cis*-regulatory elements controlling wound response overlap with *S. pennellii* (Liu et al. 2018; Jones and Vandepoele 2020).

### Rewiring gene networks as an adaptive strategy

The pattern of gene expression can change faster than the divergence of nucleotides. This may be due to the modification of TF binding sites or the emergence of *cis*-regulatory elements leading to new TF expression profiles (Winkelmüller et al. 2021). Accordingly, shifts in gene regulation provide a more flexible means of adapting to changing environmental conditions and fine-tuning genomic predisposition (Koenig et al. 2013). The evolution of GRNs has been subject to intensive debates, with 2 main hypotheses proposed to explain regulatory diversity: hub conservation, where downstream differentiation occurs, versus hub divergence, where central regulatory genes shift in function. Some studies suggest that regulatory hub genes might remain conserved (Masalia et al. 2017). However, Wei et al. (2024) emphasized the importance of hub gene divergence and subsequent network topology shifts as the key drivers of *S. chilense* adaptation to drought stress. Indeed, network re-wiring, potentially via modifying a single core gene, can significantly affect phenotypic plasticity (Koubkova-Yu et al. 2018). This may lead to highly specific responses, such as detoxification or enhanced protein localization processes, as observed in this study. A recent study by Dong et al. (2024) found evidence for GRN-rewiring by reimplementing an ancient TF, which altered the response to nutrient status in *Marchantia polymorpha.* Here, we present evidence of network rewiring leading to increased levels of QDR against a necrotrophic pathogen. Interestingly, both conserved and shared QDR-GRNs significantly enrich evolutionary younger genes dating back to Mesangiospermae–Solanales and discriminate older genes. Accordingly, we speculate that the adaptation to biotic stresses is modulated via shifts in the regulation and not adaptation via GFFEs. Following this, core genes might be subject to negative selection, but regulating core TFs and the periphery might facilitate nuanced adaptation (Fagny and Austerlitz 2021).

### Co-option of a TF might govern QDR in *S. pennellii* LA2963

Co-option/exaptation or gene duplication followed by divergence are 2 potential mechanisms driving neofunctionalization in plant-parasite interactions (Plachetzki and Oakley 2007; Macquet et al. 2022). Especially, the co-option of TFs might display a major source of new regulatory patterns in GRNs (Artur and Kajala 2021). We identified a NAC29 TF as a key modulator of QDR in *S. pennellii.* NAC TFs govern a wide array of developmental processes (e.g. flowering, cell cycle progression, and cell division) and may play a role in abiotic stress resistance (Kim et al. 2006; Nakashima et al. 2012; Zhang et al. 2018b; Gonzalez-Bayon et al. 2019; Ueda et al. 2020). Although some members of the NAP subgroup of NAC TFs might be involved in biotic stress response, only a few studies have linked them to resistance (Zhang et al. 2018a; Ma et al. 2021; Lu et al. 2024; Tominello-Ramirez et al. 2024). Moreover, it has been reported that NAC TFs might have divergent roles in *S. lycopersicum* compared with *A. thaliana* (Li et al. 2022). The potential of NAC TFs in regulating diverse, species-specific processes highlights their evolutionary plasticity and adaptability. Interestingly, we observed differential regulation of NAC29 and changes in the downstream targets. This shift of regulatory context might be due to *S. pennellii*-specific SNPs in open chromatin *cis*-regulatory elements of this gene.

By studying the phylotranscriptomics signature of resistance-associated GRNs, we can significantly enrich our understanding of QDR by integrating gene expression data with gene age (phylostratigraphy). By classifying genes into evolutionary “strata” and evaluating their expression profiles during pathogen challenge, we can assess whether older, highly conserved defense mechanisms or younger, lineage-specific expansions (perhaps newly co-opted for defense) drive quantitative resistance. We identified peaks in gene-family founder events that we can link to predicted genome expansions, such as a putative whole-genome triplication before Solanales divergence. (Clark and Donoghue 2018; Maheepala et al. 2019; Huang et al. 2023). Subsequent expansions in angiosperms (Magnoliopsida) correlate with the reported emergence of flowering-specific genes, phytohormone signaling, pathogen defense, and secondary metabolism, underscoring genome duplication's role in trait diversification (Chanderbali et al. 2016; Clark and Donoghue 2018; Maheepala et al. 2019; Bowles et al. 2020; Huang et al. 2023). Furthermore, the subsequent rise in taxonomically restricted genes within the Solanaceae may be attributed to the same genome triplication event, suggesting that such duplications drive species-specific adaptations and innovations (Clark and Donoghue 2018). Although we found evidence for species-specific gene-family expansion, we did not observe a link between taxonomically restricted genes on species level with QDR.

## Conclusion

Based on the phylotranscriptomic signature (i.e. shared copy numbers across *Solanum* species, broad basal expression patterns, and an ancient origin dating back to cellular organisms) and its unique functional role in *S. pennellii*, we propose that NAC29 has been co-opted for resistance-related functions. The parallel shifts in TF expression and downstream genes suggest that different mechanisms determine NAC29 co-option. Possibly, altered TF regulation and differentiation of TF targets might underlie strong species-specific variability (e.g. *cis*-/*trans*-regulation and/or chromatin modifications, Jones and Vandepoele 2020). We provide evidence that alterations in *cis*-elements might drive specified QDR responses. Naturally occurring loss-of-function mutants in wild *S. pennellii* accession confirm the role of NAC29 in QDR. Overall, NAC29 may serve as a prime example of how TFs can be repurposed for QDR, illustrating the broader role of TF co-option in plant adaptation.

## Materials and methods

### Plant growth conditions

We obtained germplasm from the C. M. Rick Tomato Genetics Resource Center at UC Davis (see Supplementary Table S1, TGRC UC-Davis, https://tgrc.ucdavis.edu/) and cultivated the plants in the greenhouse of the Phytopathology Department at Kiel University, Kiel, Germany. We surface-sterilized the seeds with a 2.75% hypochlorite solution. We propagated mature plants through cuttings using Chryzotop Grün 0.25% in Stender C700 substrate. We maintained the growing environment at ∼21 °C (±10 °C), 65% relative humidity, and a 16-h photoperiod. We fertilized the plants monthly using a drip irrigation system with a 1% Sagaphos Blue solution. For further methodological details, please refer to Einspanier et al. (2024), Tominello-Ramirez et al. (2024).

### Fungal growth conditions

We freshly grew the fungus *S. sclerotiorum* (1980) on potato dextrose agar (PDA) (Sigma-Aldrich) at 25 °C in the dark. We performed the inoculation using a liquid mycelium macerate, following the method described in Einspanier et al. (2024). Briefly, we incubated 100 mL of potato dextrose broth with four 1-cm pieces of fully overgrown PDA on a rotary shaker (120 rpm, 24 °C) for 4 d. After incubation, we mixed the culture using a dispenser and vacuum-filtered it through cheesecloth. We then concentrated the filtrate to an optical density at 600 nm (OD_600_) of 1 using the clear supernatant as dilution. We used fresh potato dextrose broth as a negative control and added Tween-80 as a surfactant.

### Experimental conditions

The experimental conditions of the sequencing experiment were identical to those of previous experiments conducted to measure LDT (Sucher et al. 2020; Einspanier et al. 2024). In short, we placed detached leaflets on wet tissue papers in a tray with the adaxial side up. Then, the leaves were inoculated with 10 µL of fungal mycelial mixture (OD_600_ = 1) or empty potato dextrose broth. The hood was covered and incubated at 23 °C. LED lights were used to continue the 16-h photoperiod. We sampled during the middle of lesion expansion at the same time of day, minimizing the influence of the circadian rhythm on gene expression. The specific sampling time point during mid-lesion growth was determined for each genotype based on prior high-resolution phenotyping of lesion growth dynamics (Einspanier et al. 2024). A sterile scalpel was used to sample a 2 cm × 2 cm piece covering the lesion and the surrounding tissue. Two leaf segments were pooled into 1 sample. We sampled 4 biological replicates per condition and genotype. The leaf segments were submerged in 750 µL DNA/RNA shield (Zymo Research) in ZR BashingBead Lysis Tubes (2 mm). We used the Zymo Research Quick-RNA Plant Kit for total RNA isolation following the manufacturer's protocol. We used a NanoDrop One (Thermo Fisher) and agarose gels to determine RNA quality and integrity. For RNA yield quantification, we used a Fluorometer (Promega Quantus).

### Sequencing and library preparation

We in-house prepped a Lexogen QuantSeq 3′ mRNA-Seq V2 (Lexogen, Vienna, Austria) library, following the manufacturer's guidelines. Spike-RNA was added for quality assessment, as was the PCR Add-on Kit, to determine the correct number of PCR cycles required to amplify mRNA sequences. The library was sequenced at the Competence Centre for Genomic Analysis Kiel (CCGA) on an Illumina NovaSeq 600 (Illumina, San Diego, CA, USA) with 2 × 100 bp aiming for 5 mio. reads per sample.

### Bioinformatic preprocessing

We performed a quality assessment on the raw reads using FastQC/MultiQC (Simon 2010; Ewels et al. 2016). Then, we used Cutadapt v4.8 for initial filtering and adapter trimming following recommendations from the library manufacturer (Martin 2011). This included the settings -a “polyA = A{20}” -a “QUALITY = G{20}” and custom options for adapter removal (see Online resources). Subsequently, we depleted ribosomal RNA bioinformatically using a custom pipeline (see Online resources). For this, we generated reference-rRNA sequences for all 5 species based on 45S rDNA-sequences of the 5.8S, 18S, and 25S subunits from the *A. thaliana* quality reference genome (GenBank IDs: 5.8S: AB373816.1, 25S: OK073662.1 18S: OK073663.1, Rabanal et al. 2017) and the chloroplast/mitochondrial rRNA of *S. lycopersicum* (Gene IDs: 34678306, 3950431, 3950467, 3950435, 3950433, 34678288, 34678318, 34678306). We then extracted species-specific rRNA sequences using Blast v2.13 (Camacho et al. 2009). We inflated the flanking sequences by 100 bp and extracted the final sequences using BEDTools v2.31.1 “getfasta” (Quinlan and Hall 2010). We used the following reference genomes: *S. lycopersicoides* (BioProject PRJNA727176, Powell et al. 2022), *S. chilense* (BioProject: PRJNA1210999), *S. pennellii* (BioProject: PRJEB5809, Bolger et al. 2014), *S. habrochaites* (BioProject: PRJCA008297, Yu et al. 2022), *S. pimpinellifolium* (BioProject: PRJNA607731, Wang et al. 2020), and *S. sclerotiorum* (BioProject: PRJNA348385, Derbyshire et al. 2017). Trimmed reads of each species were mapped on the respective rRNA reference using the STAR-aligner v2.7.9 with custom settings retaining only nonmapping reads (see Online resources, Dobin et al. 2013). Then, we mapped the rRNA-depleted reads against the respective reference genome using the STAR aligner with settings, following the library manufacturer's recommendations (see mapping statistics in Supplementary Table S2, Fig. S9).

We used the RNA-seq read-assisted tool GeneExt (https://github.com/sebepedroslab/GeneExt, last accessed October 2024) to extend or predict missing UTR regions in our genome annotations. By using the options --peak_perc 10 --orphan -j 16 -v 1 -m 5000, we enhanced the annotation completeness significantly (Zolotarov et al. 2023). Next, we quantified aligned sequencing reads with featureCounts v2.0.6 using custom options -s 1 -T 16 -M -t exon -g gene_id as this sequencing library allows only the quantification of expression on gene-level (Liao et al. 2014).

### Annotation and proteome

We developed a custom pipeline to improve the proteome of the 5 host plant species. First, we utilized per-species GeneExt-curated genome annotations to extract transcripts using gffread v0.12.7 and extracted protein sequences based on the longest open reading frames (ORF) with TransDecoder v5.7.1 (Pertea and Pertea 2020; Haas 2024). To retain proteins with functional significance, we employed BLASTp to identify homologous proteins in the UniProt database using the parameters -max_target_seqs 1 -evalue 1e-5. (Camacho et al. 2009; UniProt Consortium 2017). We kept only those proteins with significant UniProt matches for downstream analysis. For protein sequences that did not achieve a sufficient match in UniProt, we further assessed their homology to the ITAG4 proteome of *S. lycopersicum* (https://solgenomics.net/organism/Solanum_lycopersicum/genome/) using OrthoFinder to retain tomato-specific proteins. Next, we evaluated sequences with low identity to the tomato proteome by assigning functional annotations using PANNZER2 (Törönen and Holm 2022). We retained all sequences with a PANNZER2 positive predictive value (PPV) score greater than 40%, ensuring that only confidently annotated proteins were included. Finally, we removed duplicated sequences from the proteome FASTA files using seqkit's v0.10.0.1 rmdedub command (Shen et al. 2024). For more information on the number of filtered proteins, please see Supplementary Table S19.

### Differential gene expression analysis

We conducted a quality assessment of the expression data (see Supplementary Fig. S10) and performed differential gene expression analysis using DESeq2 v1.46.0, analyzing each species separately (Love et al. 2014). Count tables were loaded, and a DESeqDataSet object was created with a design formula including genotype-treatment interaction. After setting the treatment reference level and prefiltering genes with low counts, we defined contrast matrices for comparisons such as infected versus mock across all genotypes, within susceptible or resistant genotypes, and between resistant and susceptible genotypes under infected conditions. The lfcShrink function was applied to stabilize log fold changes, and DEGs were identified based on an absolute log_2_ fold change >1 and adjusted *P*-value ≤0.05. We quantified regulatory plasticity as the infection-induced expression change for each gene in each genotype. For this, we fit a linear mixed-effects model with infection × genotype as fixed effects and a gene-specific infection slope as a random effect. The mean estimated marginal slopes (emmeans Δlog_2_FC) per genotype were then correlated with the level of QDR.

### Phylogenomics and orthology

We performed a BUSCO analysis v5.7.0 on the curated proteomes (custom options: -m protein -l solanales_odb10) for phylogenetic tree construction (Manni et al. 2021). Subsequently, we used the busco_phylogenomics pipeline to construct species phylogenies, which we visualized with Accurate Species Tree EstimatoR (ASTER*, v1.16). We used OrthoFinder v2.5.5 to derive insight into ortholog genes among the different species. We built a central OrthoFinder project incorporating the curated proteomes of the 5 core *Solanum* species and 6 more distantly related Pentapetalae plant species using the same proteome versions as in Sucher et al. (2020). We then used the R package UpSetR v1.4.0 to visualize intersections of shared/unique orthogroups across different scales. In cases where multiple DEGs (e.g. isoforms) were detected per species and orthogroup, we selected those with the most significant and strongest differential expression.

### Coexpression networks (WGCNA)

We constructed weighted gene correlation networks with the R-package WGCNA v1.73 (Langfelder and Horvath 2007, 2008). First, we constructed a pan-species network based on orthologous genes. To ensure accurate expression comparisons, we included only single-copy orthogroups in the analysis. Genes from multicopy orthogroups were excluded because their expression cannot be unambiguously assigned to a single ortholog across species, which would confound cross-species inferences. Moreover, the presence of species-specific gene expansions or contractions in multicopy families could introduce biases in module detection and obscure conserved regulatory signals. In parallel, we generated WGCNs for each species separately, including all expressed genes. For all networks, we generated regularized log-transformed (*r*log) expression values. We analyzed both species- and OG-level networks as follows.

First, the data set was inspected for potential outliers by hierarchical clustering using the hclust() function v3.6.2 (see Supplementary Fig. S3A). To derive a soft threshold, we used the pickSoftThreshold() function. We evaluated the fit of the scale-free topology model and the mean connectivity (e.g. see Supplementary Fig. S3B). We then used a custom wrapper of the blockwiseModules() function to optimize the settings for each network separately (see Online resources, Supplementary Table S20). Additionally, we used the following custom options: maxBlockSize = nrow(datExpr), networkType=“signed hybrid,” TOMType=“signed,” minModuleSize = 30, reassignThreshold = 0, checkMissingData = F, replaceMissingAdjacencies = T. We assessed the module assignment using plotDendroAndColors() and tested its robustness by preservation testing (see Online resources, Supplementary Fig. S11).

### Module–trait relationships

We investigated per-species module–trait relationships for n modules using linear models accounting for the fixed binomial effects of *genotype* (hence, resistance phenotype) and infection. We excluded the random effect *repetition* to reduce the risk of overfitting the model, as the covariate *repetition* variance approached 0.


$$ME_{n}=Genotype+Infection+Genotype:Infection$$

For the OG network, we ranked the plant genotypes according to their LDT values (see Supplementary Table S1). This ranking was used to define an ordinal scale, allowing us to infer trait associations with MEs while accounting for species-level differences and nonlinearity. The most resistant genotype was set as the contrast reference, enabling biologically interpretable comparisons of module–trait relationships across genotypes.


$$ME_{n}=Infection+Rank$$

We extracted the model estimates to visualize the OG–module–trait relationships for easier interpretation. Accordingly, we calculated partial and nonpartial *η*^2^-values for single-species networks and corrected the *P*-values with Benjamin–Hochberg correction (Yekutieli and Benjamini 1999).


$$\eta_{\mathrm{full}}^{2}=\frac{SS_{\mathrm{effect}}}{SS_{\mathrm{total}}}$$

$$\eta_{\mathrm{partial}}^{2}=\frac{SS_{\mathrm{effect}}}{SS_{\mathrm{effect}}+SS_{\mathrm{error}}}$$

We developed a custom pipeline to identify resistance-associated WGCNA-modules in species-specific coexpression networks. First, we filtered all modules for a significant genotype × infection interaction, selecting those containing genes with divergent regulatory responses between genotypes after infection (*P* < 0.001, Supplementary Figs. S12 and S13). To reduce noise, we excluded modules with small effect sizes (*η*^2^ < 0.05). Finally, we applied preservation statistics to retain modules whose *Z*-summary scores increased in resistant genotypes under infection, indicating a more coordinated and stable expression pattern specifically in the resistant background during pathogen challenge.

To define hub genes, we used a custom pipeline based on eigengene centrality and a dynamic thresholding method which identifies inflexion points of the weight distribution (Tominello-Ramirez et al. 2024). The hub gene assignment was visually inspected and adjusted if needed (see Online resources).

### GRN analysis

We conducted a gene regulatory network analysis to uncover causal regulatory relationships among the coregulatory genes identified by WGCNA. Accordingly, we discretely predicted TFs for all species using the Transcription Factor Prediction Tool of the Plant Transcription Factor Database (Jin et al. 2017). Then, we used the R-package GENIE3 v3.20 to construct the GRN using standard settings. We used the same custom script as before to filter low-weight edges and define GRN-hub genes based on eigenvector centrality.

### GO term enrichment

To gain insights into the functional framework of the various sets of genes, we performed a GO analysis on both species-level and cross-species levels. We derived GO terms using PANNZER2 for species-level analysis and converted the results into a BiNGO-compatible format with a custom R-script. We extracted the overlapping genes and GO terms from all species for cross-species analysis. Those were collected in a consensus orthogroup-based BiNGO file. Finally, we performed GO term enrichment using the Cytoscape v3.10.3 application BiNGO v3.0.5 to interpret the functional implications of defined gene sets.

### Phylostratum analysis

We employed a robust phylostratigraphic approach to classify all transcripts according to their phylogenetic origin, carefully addressing potential biases such as homology detection failure. We used the software GenEra v1.4.2, which uses the DIAMOND algorithm v2.0.14 to align the sequences of protein-coding genes of all 5 *Solanum* species against the NCBI nonredundant database (as of September 2024). The most distant taxonomic node is considered as the age of this gene family (Sayers et al. 2020; Schoch et al. 2020; Buchfink et al. 2021; Barrera-Redondo et al. 2023; Lotharukpong et al. 2024).

### Transcript age index

Following the association of genes with their putative age, we used the R-Package myTAI v0.9.3 to quantify shifts in the TAI (Drost et al. 2018). $\mathrm{TA}I_{s}$ is defined by the number of treatments (*s*), the relative gene age of *n* genes i($ps_{i}$) and the expression level of $e_{is}$, As:


$$\mathrm{TA}I_{s}=\overset{n}{\underset{i=1}{\sum }}\;\left(\frac{\;ps_{i}\;\times e_{is}}{\sum_{i=1}^{n}\;e_{is}}\right)$$

We used normalized log_2_-transformed read counts (DESeq2 rlog()) as input-expression data.

### Transcript divergence index

We employed a *dN*/*dS*-based approach to assess evolutionary divergence at the transcript level. After extracting the longest ORFs with TransDecoder v5.7.1, we applied divergence_stratigraphy() from orthologr v0.4.2 (Drost et al. 2015). This method identifies best reciprocal hits via BLASTp, aligns them pairwise with Needleman–Wunsch (Needleman and Wunsch 1970), and converts the protein alignments to codons in PAL2NAL for *dN*/*dS* calculations (Comeron 1995; Suyama et al. 2006), using *Solanum melongena* as an outgroup (Wei et al. 2020). The resulting *dN*/*dS* values were grouped into deciles (“divergence strata”) to facilitate comparisons with phylostratigraphic data. We used these strata to compute the TDI with myTAI, where $\mathrm{TD}I_{s}$ depends on each gene's divergence stratum ($ds_{i}$) and the expression level ($e_{is}$) as


$$\mathrm{TD}I_{s}=\overset{n}{\underset{i=1}{\sum }}\;\left(\frac{ds_{i}\times ei_{s}}{\sum_{i=1}^{n}\;ei_{s}}\right)$$

### Sanger sequencing of NAC29

We sequenced the NAC29 gene (Sopen05g0003630) from 8 *S. pennellii* accessions (LA1941, LA0716, LA1809, LA1282, LA2563, LA1656, and LA1303), which had previously been screened for LDT-mediated QDR against *S. sclerotiorum* (Einspanier et al. 2024). Genomic DNA was extracted using the Zymo Research Quick-DNA Plant Miniprep Kit according to the manufacturer's protocol. Gene fragments were amplified by PCR using specific primers (see Supplementary Table S21) and sequenced using Sanger sequencing technology (Eurofins). Sanger sequencing reads were quality-trimmed and mapped to the reference genome in Geneious Prime. CDSs were used for multiple sequence alignment (MUSCLE) and maximum likelihood tree construction.

### NAC29 promoter analysis

To characterize diversity in the NAC29 promoter across different host plants, we extracted 2-kb upstream regions from 8 species, including *A. thaliana*, *S. lycopersicum*, and *S. melongena*. Multiple sequence alignment was performed using mVISTA with the MLAGAN algorithm to identify conserved noncoding sequences (CNS), defined as regions with ≥70% sequence identity in 100-bp sliding windows. CNS overlapping with open chromatin peaks (based on ATAC-seq data from *S. lycopersicum*, GEO Series accession number GSE164297) were defined as accessible promoter regions and used for detailed sequence comparison to assess species-specific variation.

### Statistics and visualization

We used the programming language R v4.4.0 in RStudio for statistical analysis. In particular, we used packages of the tidyverse v2.0.0, such as tidyr v1.3.1, stringr v1.5.1, and dplyr v1.1.4. All figures were prepared using ggplot2 v3.5.1 and curated for publishing in Inkscape.

### Online resources

Detailed code, including all scripts, documentation and environment information is available at the following GitHub repository: github.com/PHYTOPatCAU/SolanumPhylotranscriptomics.

### Accession numbers

Sequence data from this article can be found at the Arabidopsis Genome Initiative (TAIR) or at the Sol Genomic Network (see Supplementary Table S17, Data Sets 1 to 6).

## Supplementary Material

koaf233_Supplementary_Data

## Data Availability

Sequence data and processed read counts from this article are available in the NCBI GEO repository under accession number GSE288242 (see Supplementary Table S22).

## Supplementary data

The following materials are available in the online version of this article.

**Supplementary Figure S1.** Relationship of read count and number of assigned DEGs.

**Supplementary Figure S2.** Amount of infection-induced differentially expressed orthogroups.

**Supplementary Figure S3.** Sample evaluation for the OG-based WGCNA.

**Supplementary Figure S4.**  *dN*/*dS* ratios contrasting significantly between overlapping versus unique versus all genes from the resistance modules.

**Supplementary Figure S5.** Analysis of the NAC29 CDS reveals interspecific and intraspecific diversity.

**Supplementary Figure S6.** NAC29 promoter analysis.

**Supplementary Figure S7.** Expression profiles of resistance-associated TFs across *Solanum.*

**Supplementary Figure S8.** Phylostratum enrichment analysis per species of the 3 focal TFs.

**Supplementary Figure S9.** Mapping statistics of *S. sclerotiorum* reads.

**Supplementary Figure S10.** Basic differential gene expression analysis of all 5 tomato species.

**Supplementary Figure S11.** WGCNA dendrogram showing network topology and module assignment of each species.

**Supplementary Figure S12.** MEs of the *S. pennellii* network.

**Supplementary Figure S13.** MEs of the *S. lycopersicoides* network.

**Supplementary Table S1.** Disease-resistance-parameters of the different accessions.

**Supplementary Table S2.** Mapping statistics.

**Supplementary Table S3.** Infection-induced number of DEGs.

**Supplementary Table S4.** Information on the putative function of *S. pennellii* sDEOGs.

**Supplementary Table S5.** Information on the putative function of *S. habrochaites* sDEOGs.

**Supplementary Table S6.** Information on the putative function of *S. lycopersicoides* sDEOGs.

**Supplementary Table S7.** Information on the putative function of *S. chilense* sDEOGs.

**Supplementary Table S8.** Information on the putative function of *S. pimpinellifolium* sDEOGs.

**Supplementary Table S9.** Function and ITAG4-Gene IDs of the 16 induced cDEOGs (infection-induced, conserved, differentially expressed orthogroups).

**Supplementary Table S10.** Position of icDEOG in the *S. pennellii* GRN and WGCN.

**Supplementary Table S11.** Full and partial *η*^2^ of OG-WGCNA modules.

**Supplementary Table S12.**  *S. pennellii* module functional association.

**Supplementary Table S13.**  *S. lycopersicoides* module functional association.

**Supplementary Table S14.**  *S. pennellii* resistance module preservation in *S. lycopersicoides* network.

**Supplementary Table S15.**  *S. lycopersicoides* resistance module preservation in *S. pennellii* network.

**Supplementary Table S16.** TDI of the *S. pennellii* and *S. lycopersicoides* resistance modules, the overlapping- and unique subset across different experimental conditions.

**Supplementary Table S17.** Functional characterization of putatively QDR-associated candidate genes identified using GRN/WGCNA analysis in *S. lycopersicoides* and *S. pennellii*.

**Supplementary Table S18.**  *dN*/*dS* and phylostratigraphy analysis of the main resistance TF in *S. lycopersicoides* and *S. pennellii.*

**Supplementary Table S19.** Number of proteins after the respective filtering steps.

**Supplementary Table S20.** WGCNA-settings for network-construction, all network constructed using blockwiseModules.

**Supplementary Table S21.** Primer sequences used in this study.

**Supplementary Table S22.** GEO accession numbers of all samples.

**Supplementary Data Set 1.** Protein sequence alignment of NAC29 orthogroup (OG0005445) across all species analyzed in this study.

**Supplementary Data Set 2.** Neighbor-joining consensus tree of aligned NAC29 orthogroup (OG0005445) protein sequences.

**Supplementary Data Set 3.** Alignment of *S. pennellii* NAC29 sequences across 7 genotypes.

**Supplementary Data Set 4.** Neighbor-joining tree of aligned *S. pennellii* NAC29 sequences across 7 genotypes.

**Supplementary Data Set 5.** Sequence alignment of the first *cis*-regulatory region of NAC29 across 6 *Solanum* species.

**Supplementary Data Set 6.** Sequence alignment of the second *cis*-regulatory region of NAC29 across 6 *Solanum* species.
