# Supplementary material for: Co-option of transcription factors drives evolution of quantitative disease resistance against a necrotrophic pathogen
Source: Plant Cell. 2025 Sep 30;37(10):koaf233. doi: 10.1093/plcell/koaf233 (PMC12527349; doi:10.1093/plcell/koaf233)
Supplement: koaf233_Supplementary_Data [file koaf233_supplementary_data.zip › supplementary_figures.pdf]

**Supplementary Figures for:**

# Co-option of transcription factors drives evolution of quantitative disease resistance against a necrotrophic pathogen

**Einspanier, S.[1]; Tominello-Ramirez, C[2,1].; Delplace, F.[3]; Stam, R.[1*]**

1 Department of Phytopathology and Crop Protection, Institute of Phytopathology, Faculty of Agricultural and Nutritional Sciences, Christian Albrechts University, Kiel, Germany

2 Department of Nutriinformatics, Institute of Human Nutrition and Food Sciences, Faculty of Agricultural and Nutritional Sciences, Christian Albrechts University, Kiel, Germany

3 Laboratoire des Interactions Plantes Microorganismes Environnement (LIPME), INRAE, CNRS, Castanet Tolosan Cedex, France

* Address correspondence to: remco.stam@phytomed.uni-kiel.de

**Short title: Co-option drives QDR against necrotrophic pathogen**

---

The authors responsible for distribution of materials integral to the findings presented in this article in accordance with the policy described in the Instructions for Authors (https://academic.oup.com/plcell/pages/General-Instructions) are: Severin Einspanier (s.einspanier@phytomed.uni-kiel.de) and Remco Stam (remco.stam@phytomed.uni-kiel.de).

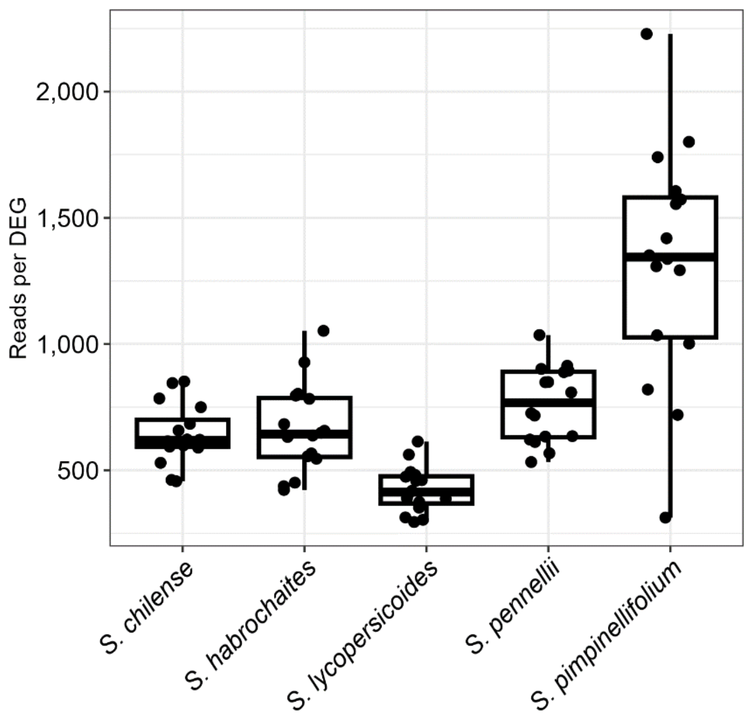


**Suppl. Figure S 1: Relationship of read count and number of assigned DEGs.** We calculated the ratio based on the number of DEGs (contrast: infection vs. mock) per genotype and normalised it against the individual reads per sample (as represented by the dots). The centre line indicates the median, the box shows the interquartile range (IQR), and the whiskers extend to the largest and smallest values within 1.5 × IQR from the hinges.

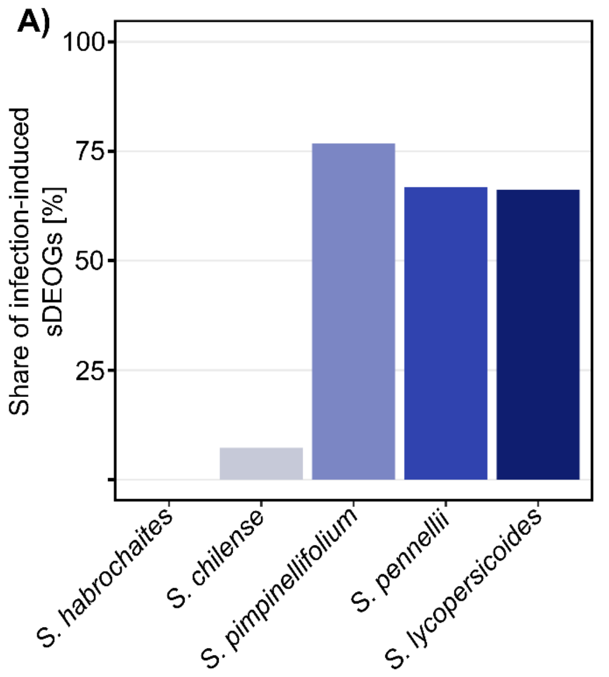


B)

| Infection-induced in: | Number of cDEOGs |
|---|---|
| no species | 1 |
| one species | 11 |
| two species | 50 |
| three species | 96 |
| four species | 65 |
| five species | 16 |

**Suppl. Figure S 2: Amount of infection-induced differentially expressed orthogroups.**
**A)** species-specific sDEOGs and **B)** cDEOGs.

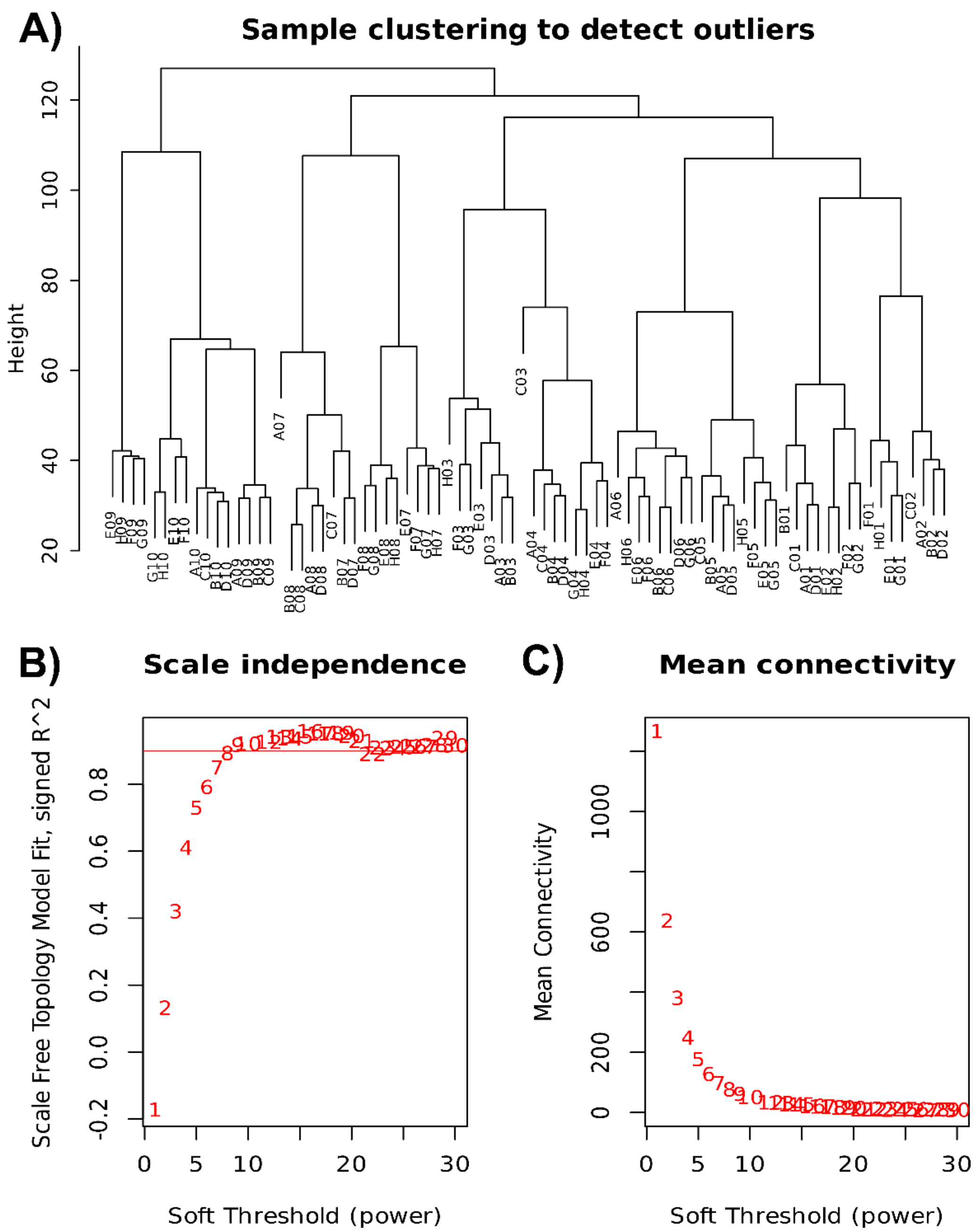


**Suppl. Figure S 3: Sample evaluation for the OG-based WGCNA.**
**A)** Hierarchical clustering of all samples used in the study. **B)** Selection of the Soft power threshold for WGCNA network construction on the OG-data set. The red line indicates the signed $R^2$ threshold of 0.85. The SFT threshold was selected based on the scale-free topology model's plateau and mean connectivity (**C**).

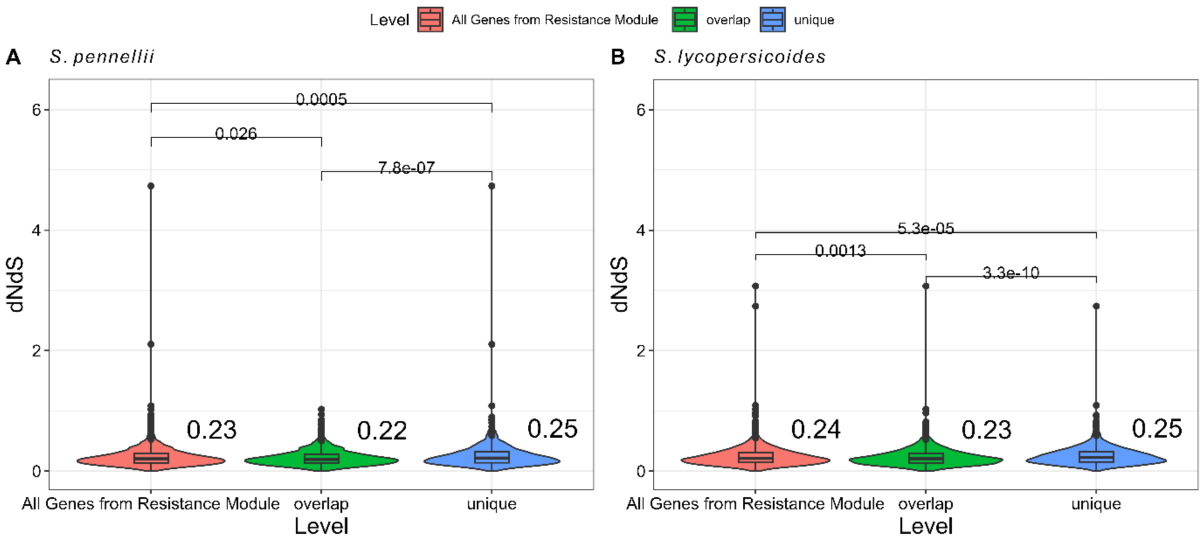


**Suppl. Figure S 4: dNdS ratios contrasting differ significantly between overlapping vs. unique vs. all genes from the resistance modules.** The violin plots indicate the distribution of the ratio of dNdS (ratio of synonymous to non-synonymous mutations) values on **A)** *S. pennellii* and **B)** *S. lycopersicoides*. Genes from different gene sets were compared: genes from the putative resistance modules, unique and genes that overlap between those modules of both species. Statistical significance was determined using the Wilcoxon test. Numbers next to the violin charts represent the mean.

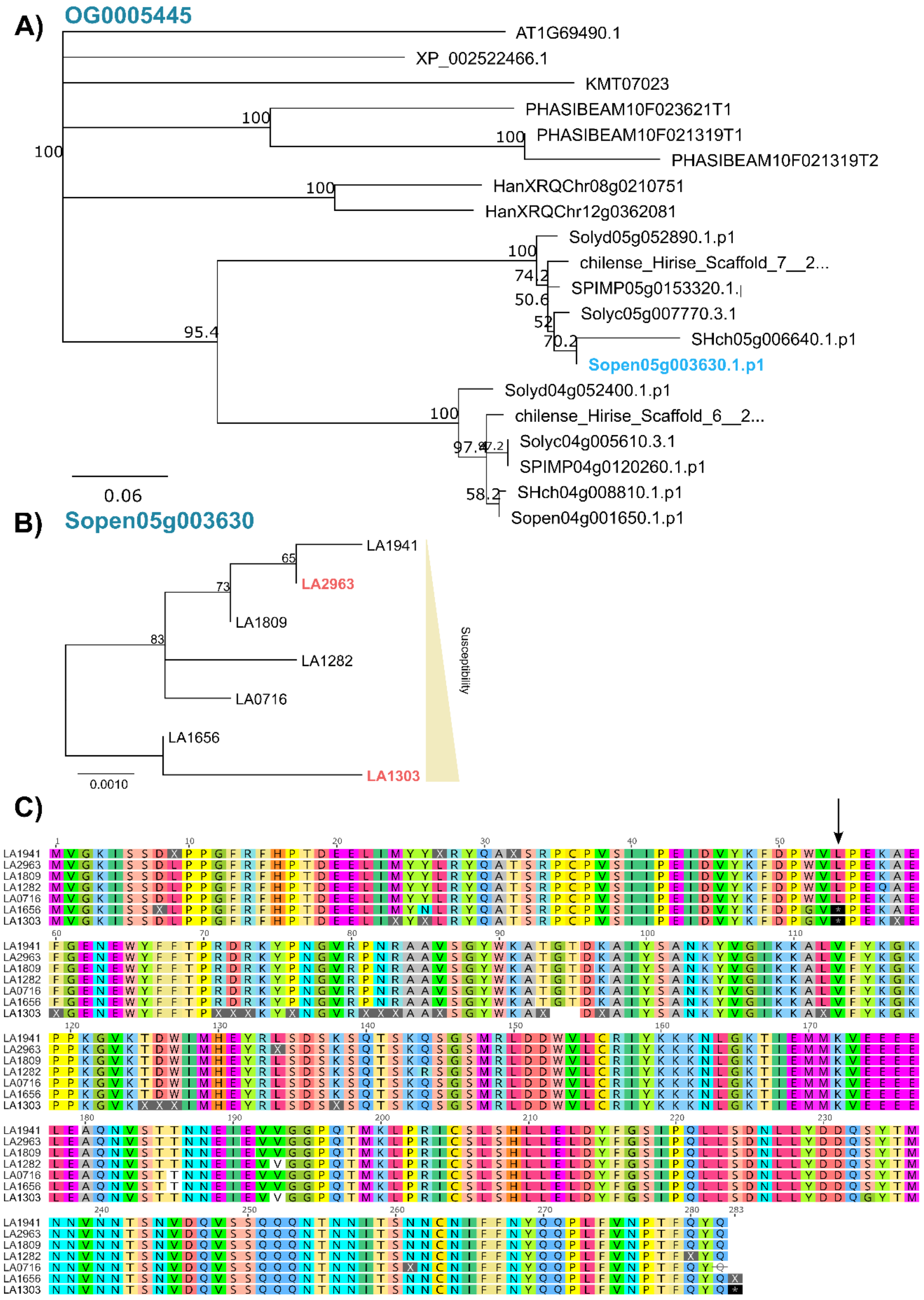


**Suppl. Figure S 5:Analysis of the NAC29 coding sequence reveals inter- and intraspecific diversity. A)** Phylogenetic tree of all members of the NAC29-like orthogroup (OG0005445). The focal *S. pennellii* allele is highlighted in blue. **B)** Phylogenetic tree based on CDS sequences of eight *S. pennellii* genotypes, spanning a gradient of LDT-mediated resistance (see Einspanier et al. 2024). The genotypes used in this study are in red font. The scale bars indicate the unit in substitutions/site. **C)** Codon-based protein sequence alignment of the eight *S. pennellii* genotypes showing non-synonymous variation. The arrow indicates premature stop codons found in two highly susceptible genotypes.

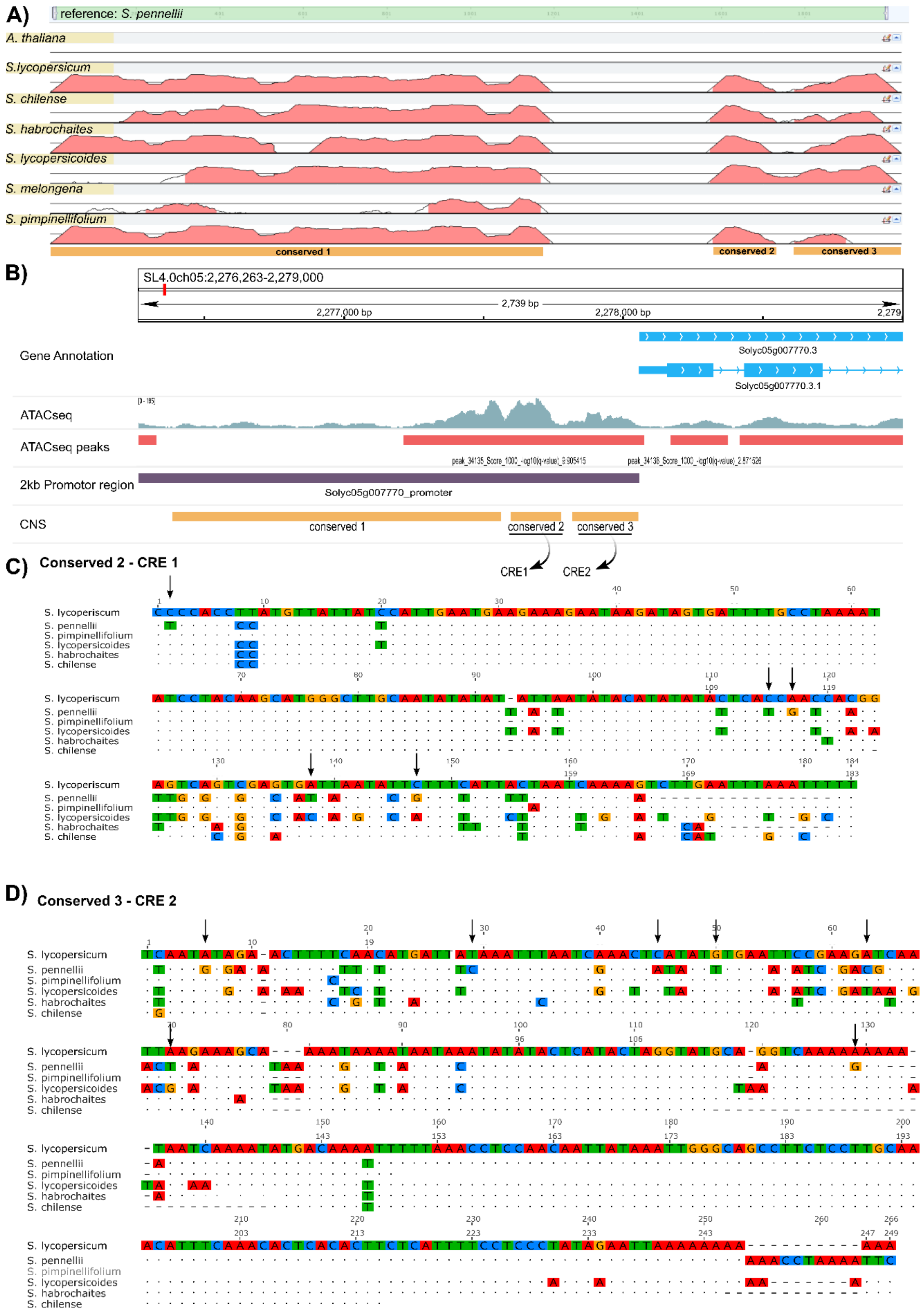


**Suppl. Figure S 6: NAC29 Promoter Analysis.** We performed phylogenetic footprinting **(A)** combined with ATAC-seq data from https://doi.org/10.1016/j.cell.2021.02.001 to identify cis-regulatory elements (CREs) within regions of open chromatin upstream of NAC29. **(B)** Two candidate promoter regions were identified based on sequence conservation and chromatin accessibility. **(C, D)** Multiple sequence alignments of these promoter regions across six Solanum species. SNPs unique to S. pennellii are indicated with arrows, highlighting species-specific regulatory variation.

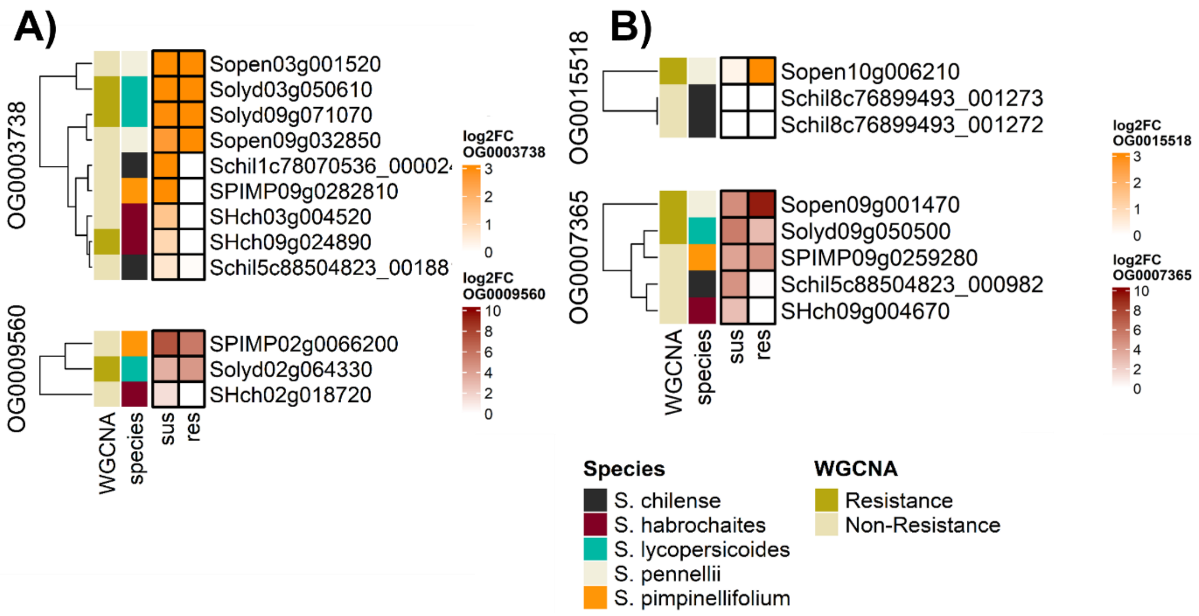


**Suppl. Figure S 7: Expression profiles of resistance-associated transcription factors across Solanum species using orthogroups.** Transcription factors with a potential role in QDR were identified in **A)** *S. pennellii* and **B)** *S. lycopersicoides*. The expression of each gene within the respective orthogroup was visualised across varying levels of QDR (susceptible | resistant), with colour coding indicating the respective species and the assignment of each TF to its species-specific resistance module.

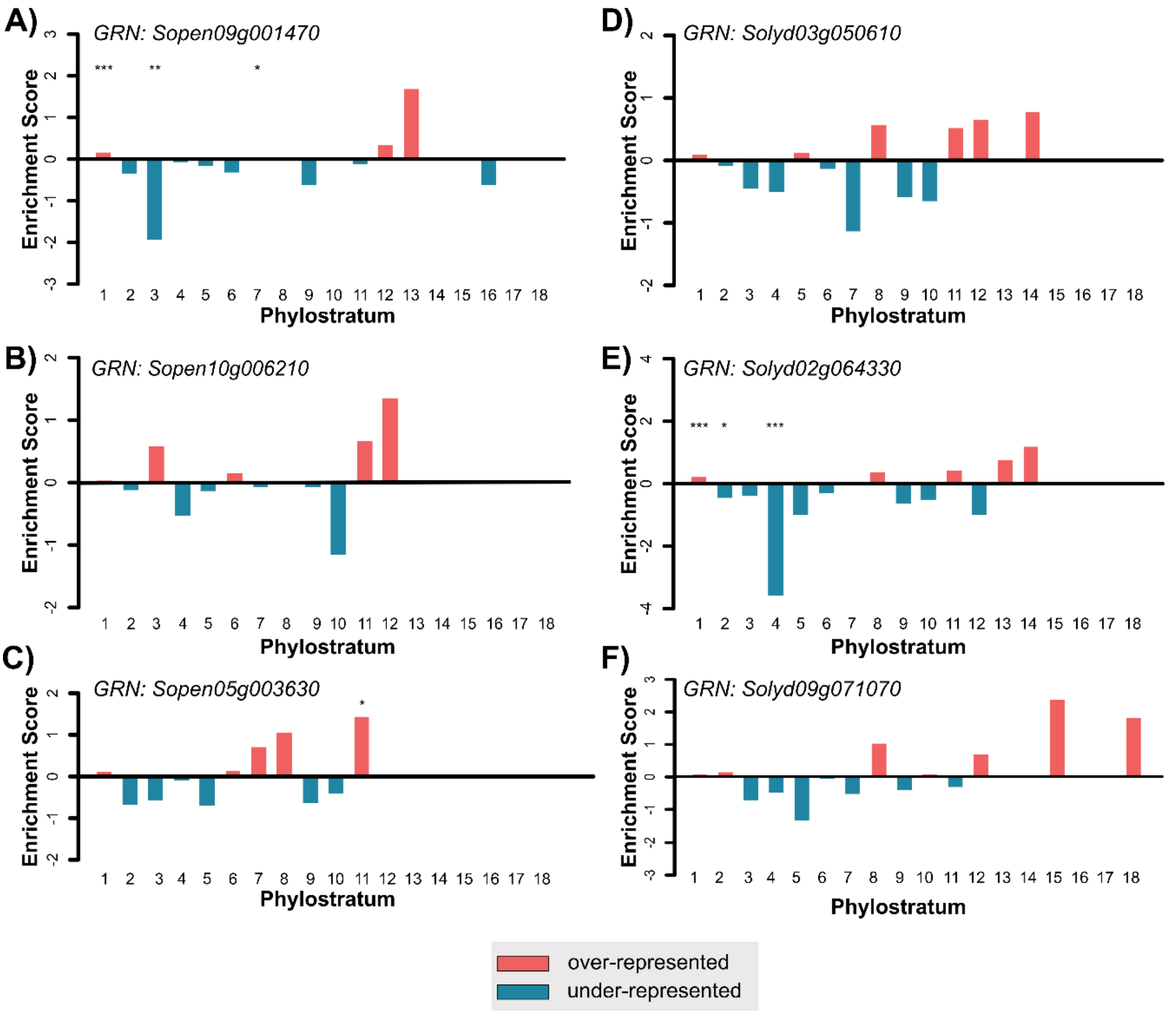


**Suppl. Figure S 8: Phylostratum enrichment analysis per species of the three focal transcription factors.** We performed phylostratum enrichment analysis on the gene-regulatory networks downstream of three TFs on *S. pennellii* **(A-C)** and *S. lycopersicoides* **(C-E).** The enrichment score is indicated by the y-axis, and the respective phylostrata (cellular organisms till solanum) are located on the x-axis. Stars indicate the significance level of the respective enrichment after Fisher's exact test and BH FDR correction.

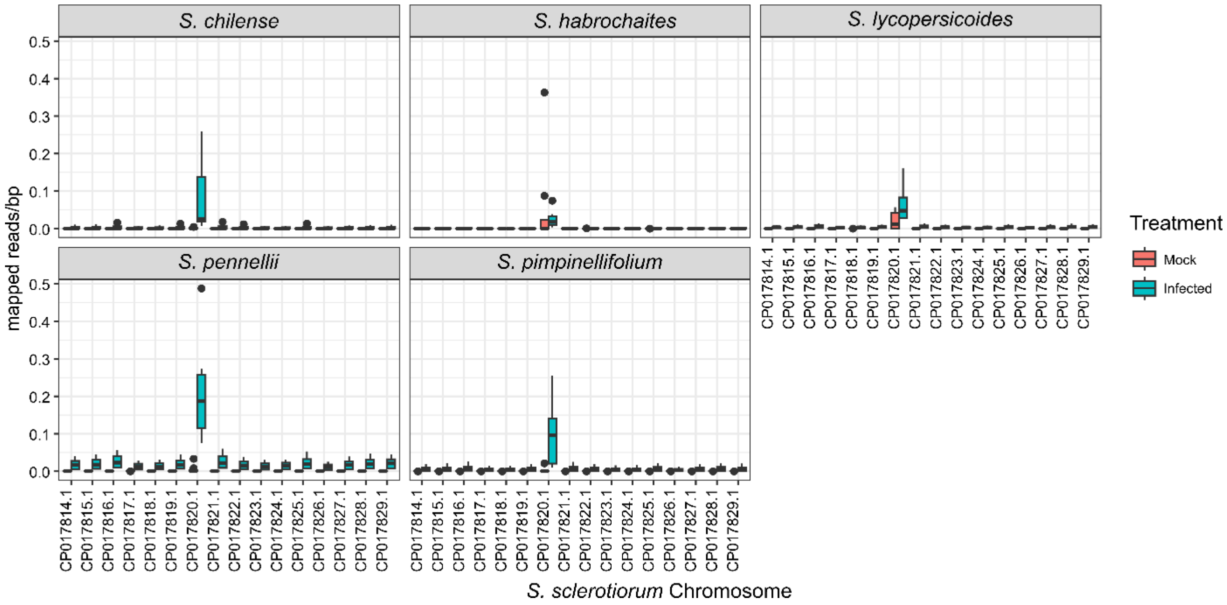


**Suppl. Figure S 9: Mapping statistics of *S. sclerotiorum* reads.** The number of mapped reads (indicated as boxplots) is unevenly distributed across the genome in all host species. The centre line indicates s the median, and the box shows the interquartile range. Sclerotinia-infected samples are indicated by a blue box, mock-samples in red.

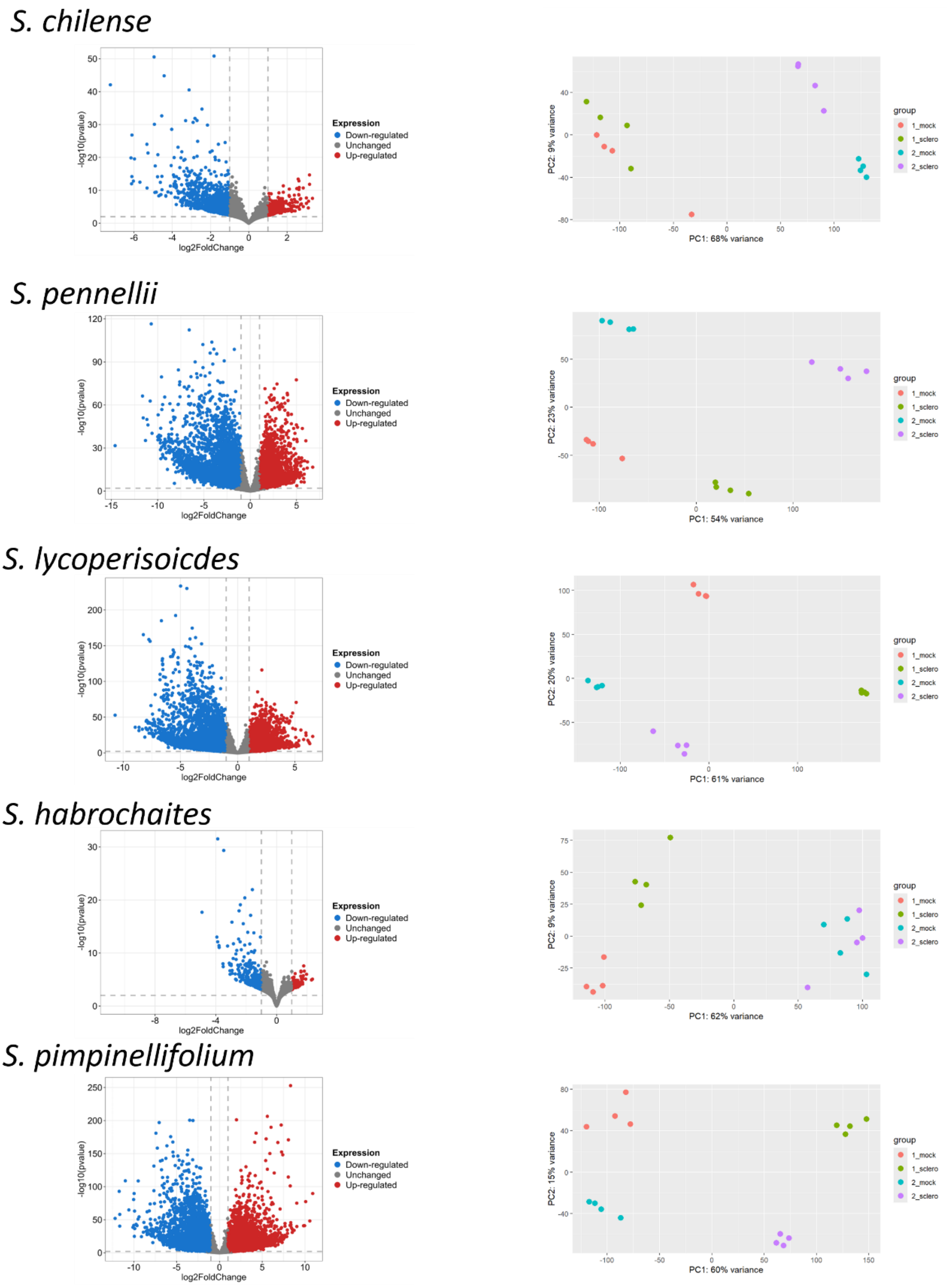


**Suppl. Figure S 10: Basic Differential Gene Expression Analysis of all five tomato species.** The volcano plots show log2 foldchange against the p-value of all five tomato species (contrast: Infected conditions res.-sus. genotype). Upregulated genes (red) are compared with downregulated genes (blue). The principal component analysis shows a clear separation of genotypes and treatments. Dots represent individual samples, with the colour defining the experimental treatment (1-susceptible, 2-resistant, mock and infected).

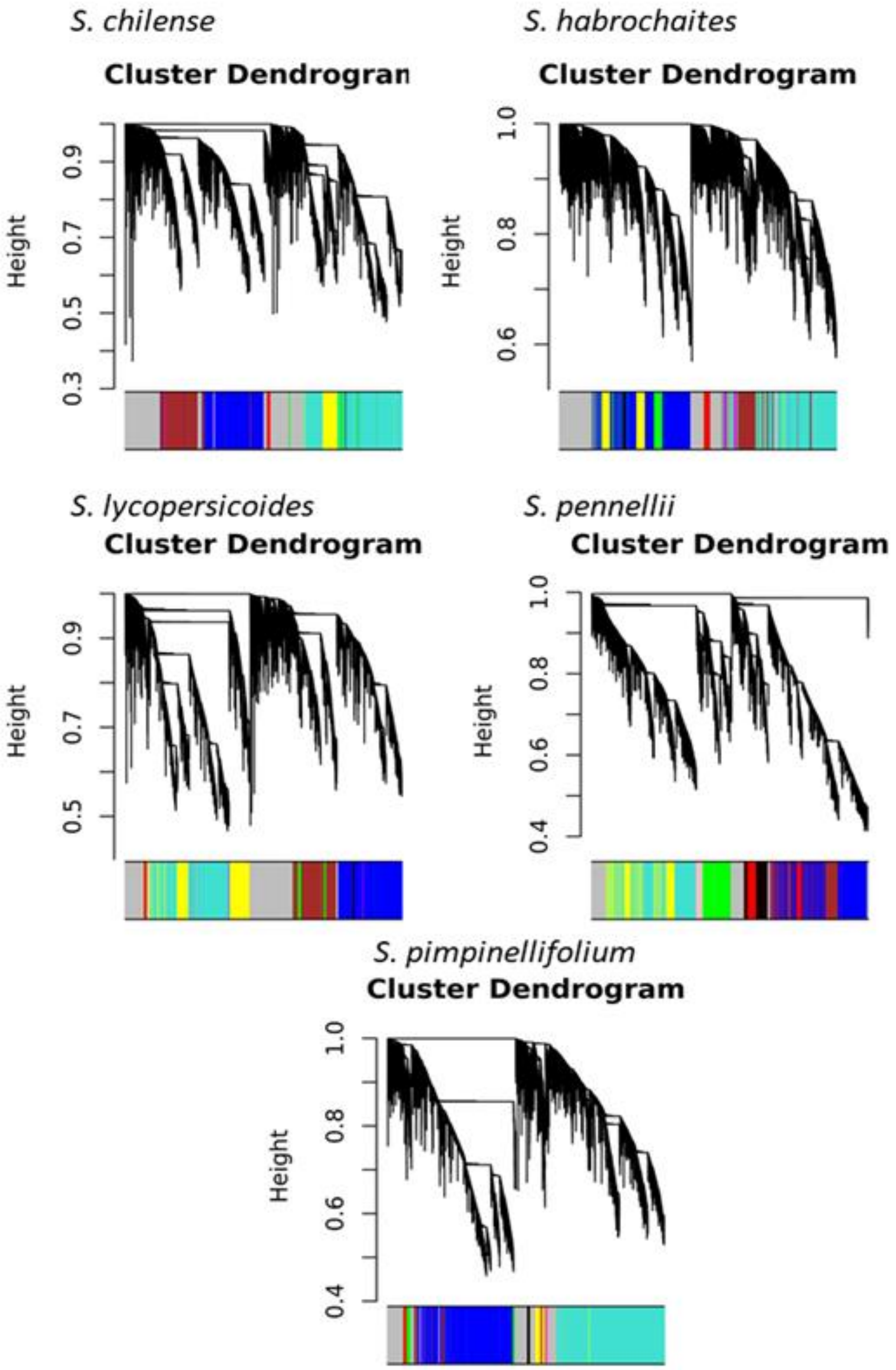


**Suppl. Figure S 11: WGCNA dendrogram showing network topology and module assignment of each species.** Each gene is represented as a branch of the dendrogram, positioned according to its co-expression with other genes. The colored ribbon beneath the dendrogram indicates module membership. Branch height reflects the degree of dissimilarity in expression profiles, which underlies module assignment.

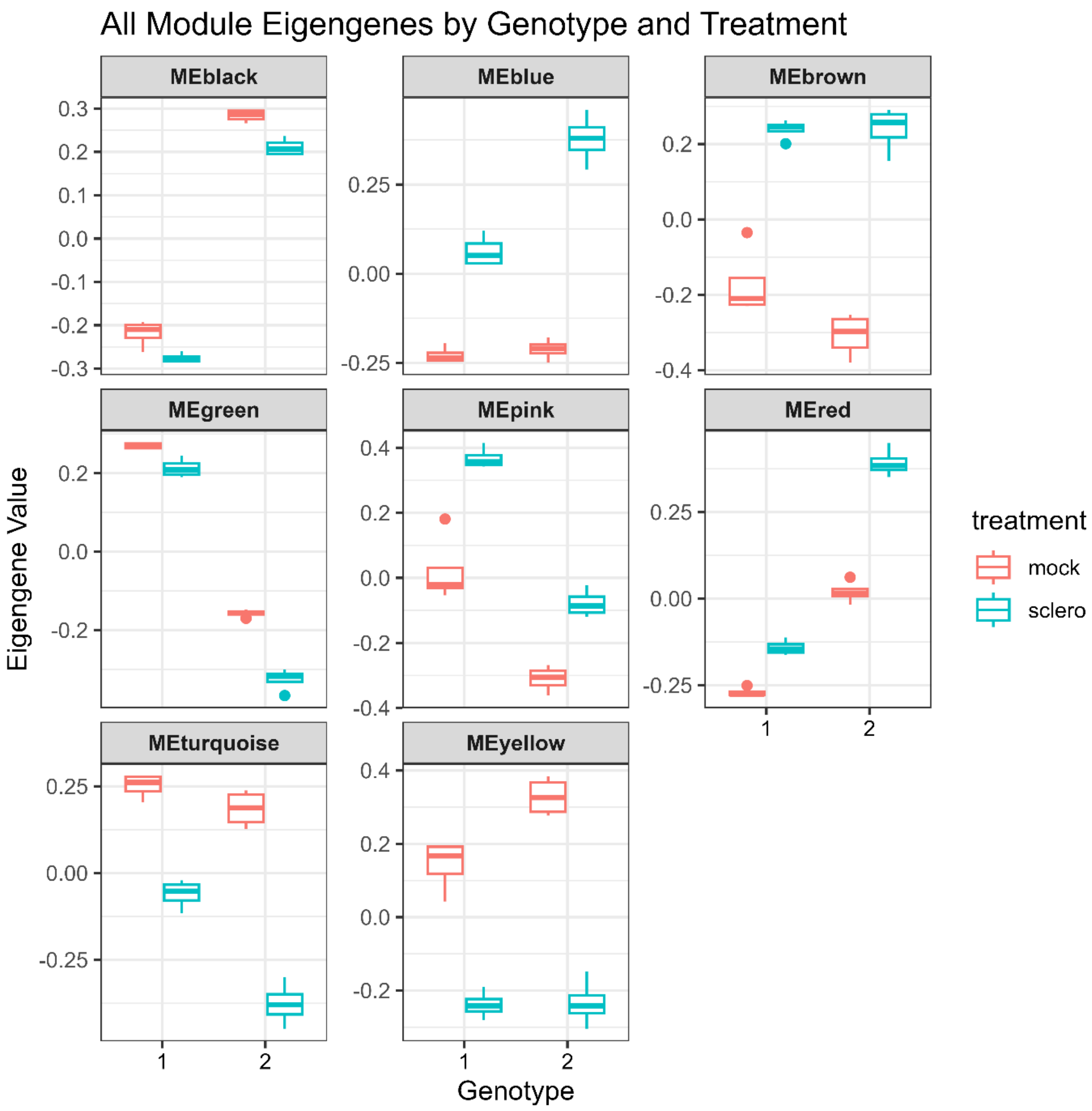


**Suppl. Figure S 12: Module Eigengenes of the *S. pennellii* network.** The individual facets define coexpression modules assigned by the WGCNA. The Y-axis denominates the module eigengenes (MEs) of the two genotypes (1 susceptible, 2 resistant). The colour indicates experimental treatment. Boxplots summarise four independent samples. The centre line indicates the median, the box shows the interquartile range (IQR), and the whiskers extend to the largest and smallest values within 1.5 × IQR from the hinges.

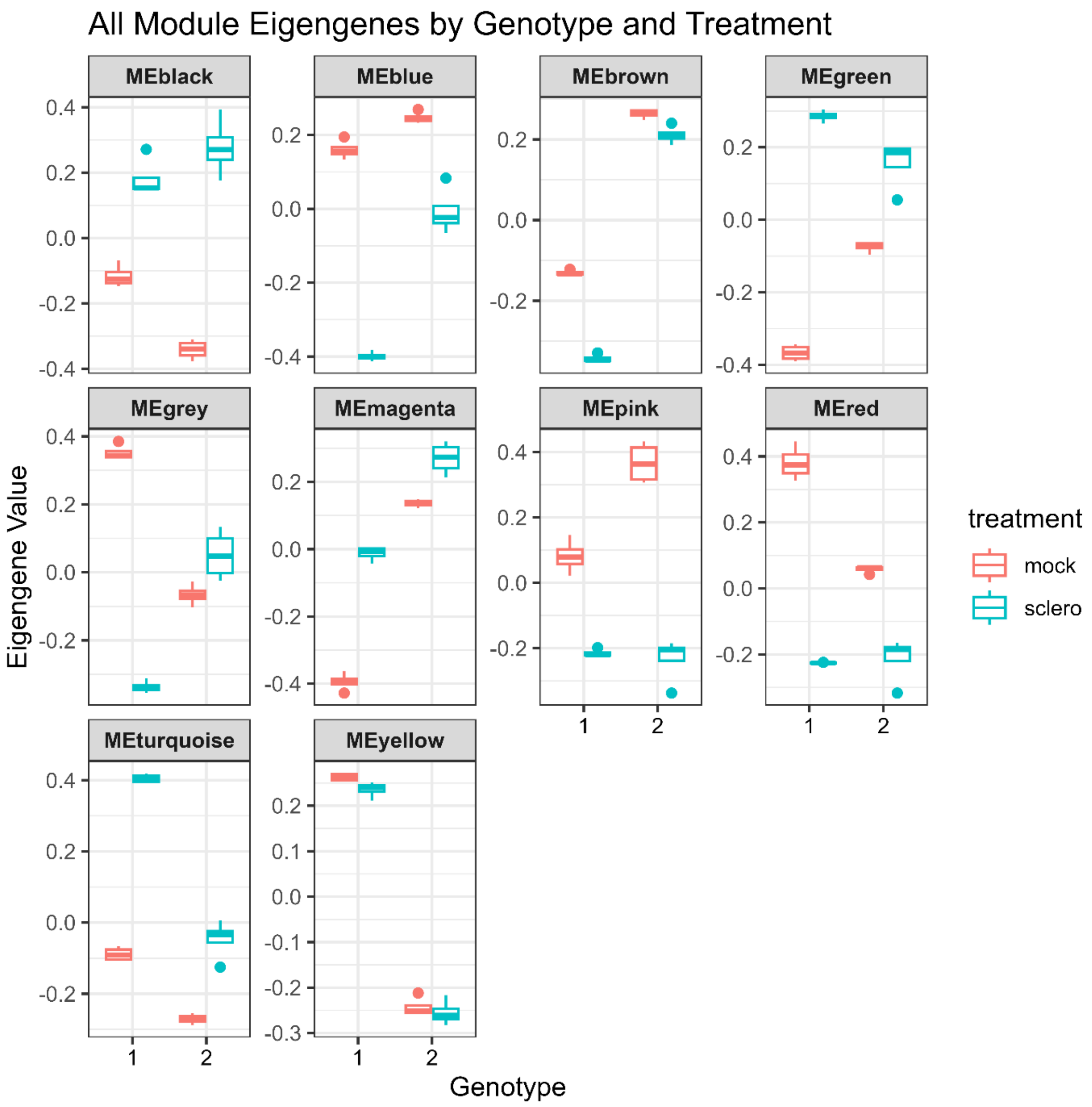


**Suppl. Figure S 13: Module Eigengenes of the *S. lycopersicoides* Network.** The individual facets define coexpression modules assigned by the WGCNA. The Y-axis denominates the module eigengenes (MEs) of the two genotypes (1 susceptible, 2 resistant). The colour indicates experimental treatment. The boxplots summarise four independent samples. The centre line indicates the median, the box shows the interquartile range (IQR), and the whiskers extend to the largest and smallest values within 1.5 × IQR from the hinges.
